# Sublytic C5b-9 induces glomerular mesangial cell proliferation via ERK1/2-dependent SOX9 phosphorylation and acetylation by enhancing Cyclin D1 in rat Thy-1 nephritis

**DOI:** 10.1038/s12276-021-00589-9

**Published:** 2021-04-02

**Authors:** Mengxiao Xie, Zhijiao Wu, Shuai Ying, Longfei Liu, Chenhui Zhao, Chunlei Yao, Zhiwei Zhang, Can Luo, Wenbo Wang, Dan Zhao, Jing Zhang, Wen Qiu, Yingwei Wang

**Affiliations:** 1 Department of Immunology, and Key Laboratory of Immunological Environment and Disease, Nanjing Medical University, 101 Longmian Road, Nanjing, Jiangsu 211166 China; 2 Department of Laboratory Medicine, The First Affiliated Hospital of Nanjing Medical University, 300 Guangzhou Road, Nanjing, Jiangsu 210029 China; 3 Department of Central Laboratory, The Affiliated Huaian No. 1 People’s Hospital, Nanjing Medical University, One West Huanghe Road, Huai’an, Jiangsu 223300 China; 4 Department of Oncology, The First Affiliated Hospital of Nanjing Medical University, 300 Guangzhou Road, Nanjing, Jiangsu 210029 China; 5 Department of Nephrology, The First Affiliated Hospital of Nanjing Medical University, 300 Guangzhou Road, Nanjing, Jiangsu 210029 China; 6 Key Laboratory of Antibody Technology of Ministry of Health, Nanjing Medical University, Nanjing, Jiangsu 211166 China

**Keywords:** Complement cascade, Post-translational modifications

## Abstract

Glomerular mesangial cell (GMC) proliferation is a histopathological alteration in human mesangioproliferative glomerulonephritis (MsPGN) or in animal models of MsPGN, e.g., the rat Thy‐1 nephritis (Thy-1N) model. Although sublytic C5b-9 assembly on the GMC membrane can trigger cell proliferation, the mechanisms are still undefined. We found that sublytic C5b-9-induced rat GMC proliferation was driven by extracellular signal‐regulated kinase 1/2 (ERK1/2), sry-related HMG-box 9 (SOX9), and Cyclin D1. Here, ERK1/2 phosphorylation was a result of the calcium influx-PKC-α-Raf-MEK1/2 axis activated by sublytic C5b-9, and Cyclin D1 gene transcription was enhanced by ERK1/2-dependent SOX9 binding to the Cyclin D1 promoter (−582 to −238 nt). In addition, ERK1/2 not only interacted with SOX9 in the cell nucleus to mediate its phosphorylation at serine residues 64 (a new site identified by mass spectrometry) and 181 (a known site), but also indirectly induced SOX9 acetylation by elevating the expression of general control non-repressed protein 5 (GCN5), which together resulted in Cyclin D1 synthesis and GMC proliferation. Moreover, our in vivo experiments confirmed that silencing these genes ameliorated the lesions of Thy‐1N rats and reduced SOX9 phosphorylation, acetylation and Cyclin D1 expression. Furthermore, the renal tissue sections of MsPGN patients also showed higher phosphorylation or expression of ERK1/2, SOX9, and Cyclin D1. In summary, these findings suggest that sublytic C5b-9-induced GMC proliferation in rat Thy-1N requires SOX9 phosphorylation and acetylation via enhanced Cyclin D1 gene transcription, which may provide a new insight into human MsPGN pathogenesis.

## Kidney disease: Understanding why cells overproliferate

Researchers have clarified the mechanism behind a kidney disease called mesangioproliferative glomerulonephritis (MsPGN), which causes certain kidney cells to overproliferate. In MsPGN, cells called glomerular mesangial cells (GMCs) overproliferate, causing kidney lesions, decreased function, and kidney failure. The mechanism at the molecular level has been unclear. A team led by Yingwei Wang at Nanjing Medical University in China investigated what leads GMCs to proliferate, using a rat animal model. They found that the transcription factor protein SOX9, often implicated in cell proliferation, is both phosphorylated and acetylated, enhancing its transcriptional activities and permitting it to activate other molecules that control the cell cycle, leading to overproliferation of GMCs. Elucidating the disease mechanism in this animal model may help to understand the disease in humans, and identify therapeutic targets.

## Introduction

Human mesangioproliferative glomerulonephritis (MsPGN) is a primary glomerular disease with a high incidence^1,2^. Because the pathological feature of MsPGN is glomerular mesangial cell (GMC) proliferation and extracellular matrix (ECM) accumulation, which finally causes renal failure in patients^1,2^, it is very important to elucidate the pathogenic mechanisms (such as that of GMC proliferation) of this kidney disease to prevent and treat human MsPGN in the future. Currently, although many studies have found that complement C5b-9 can be deposited in the glomeruli of MsPGN patients^3–6^, the role and mechanism of C5b-9 in GMC proliferation are still unknown. Rat Thy-1 nephritis (Thy-1N) is an animal model used to study MsPGN^7–10^. Our previous reports have demonstrated that sublytic C5b-9 stimulation can induce GMC proliferation^9,11^, but the mechanism of sublytic C5b-9‐triggered proliferation in Thy‐1N rats remains unclear.

Cell proliferation is regulated by cell cycle progression. Cyclin D1 is a member of the Cyclin family that shifts cells from G1 to S phase by interacting with cyclin-dependent kinases 4 and 6^12^. Our early experiments showed upregulation of Cyclin D1 expression both in GMCs treated with sublytic C5b-9 (in vitro) and in the renal tissues of Thy‐1N rats (in vivo). However, the role of Cyclin D1 in GMC proliferation and the mechanism underlying its upregulation upon sublytic C5b-9 treatment are unexplored.

Gene expression is a process modulated by specific transcription factors^13–15^. Sry-related high mobility group protein 9 (SOX9) is a transcription factor belonging to the SRY-related HMG domain gene family^16^. Substantial evidence has confirmed that SOX9 promotes cell proliferation by regulating target genes^17–20^. Since we have demonstrated SOX9 overexpression both in vitro and in vivo, whether this overexpression induces Cyclin D1 to promote GMC proliferation in Thy-1N rats and the mechanism underlying such a process need to be clarified.

ERK1 and ERK2 are two analogous serine/threonine (Ser/Thr) kinases that phosphorylate protein substrates^21^. ERK1/2 not only regulates gene expression to induce cell proliferation upon phosphorylating substrates such as c-Fos and c-Jun^22–24^, but also fosters the acetylation of the transcription factor NFAT-c1 to elevate gene transcription^25^. Given that SOX9 can be modified at the posttranslational level, such as phosphorylation or acetylation^26,27^, and our preliminary experiments also displayed high levels of phosphorylated ERK1/2 (p-ERK1/2) and SOX9 as well as its phosphorylated and acetylated forms, it is worth ascertaining whether ERK1/2 induces and/or modifies SOX9 to promote Cyclin D1 synthesis for GMC proliferation after exposure to sublytic C5b-9 attack in Thy-1N rats.

In this investigation, we demonstrated that phosphorylated ERK1/2 (p-ERK1/2), SOX9, and Cyclin D1 were all essential for GMC proliferation induced by sublytic C5b-9. Mechanistic exploration revealed that sublytic C5b-9 formation on rat GMCs increased calcium influx, leading to the activation of the protein kinase C-α (PKC-α)-Raf-MEK1/2-ERK1/2 axis, which enabled SOX9 to occupy the Cyclin D1 promoter, leading to Cyclin D1 gene transcription. More importantly, sublytic C5b-9 triggered ERK1/2 interaction with SOX9 in the GMC nucleus, followed by SOX9 phosphorylation at Ser64 (a newly identified site) or Ser181 (a known site) and its acetylation via GCN5 upregulation by ERK1/2. Here, SOX9 phosphorylation not only augmented its acetylation and nuclear expression but also promoted Cyclin D1 transcription and GMC proliferation, whereas SOX9 acetylation had no effect on its phosphorylation. Furthermore, our in vivo results confirmed that silencing of these genes decreased SOX9 phosphorylation, acetylation and Cyclin D1 production as well as Thy-1N lesions. In addition, we also demonstrated that there is higher phosphorylation or expression of ERK1/2, SOX9, and Cyclin D1 in the renal tissues of MsPGN patients.

## Materials and methods

### Animals, cell line, and reagents

Male Sprague-Dawley rats (180–200 g) were purchased from the Animal Core Facility of Nanjing Medical University. All animal experiments were performed in compliance with the Guide for the Care and Use of Laboratory Animals and were approved by the Institutional Animal Care and Use Committee of Nanjing Medical University. Rat GMCs were provided by the China Center for Type Culture Collection (Wuhan, China). Normal human serum (NHS) collected from healthy donors was used to supply complement, and heat-inactivated serum (HIS) was obtained by incubating NHS at 56 °C for 30 min. Human C6-deficient serum (C6DS, A323) was purchased from Complement Technology (USA). Recombinant human C6 (12426-H08H) was obtained from Sino Biological Inc (P.R. China.). Rabbit polyclonal anti-Thy-1 ab and normal rabbit serum (NRS, 36117ES03), together with anti-HA affinity gel (20586ES03), were prepared in our laboratory^11^ or purchased from Yeasen (P.R. China.). Cell Counting Kit-8 (CK04) and Cell-Light™ EdU Apollo^®^567 (C10310-1) were supplied by Dojindo (Japan) and RiboBio (P.R. China.), respectively. HiScript II Q RT SuperMix for qPCR (R222-01), 2× Taq Master Mix (P212-01), and ClonExpress Ultra One Step Cloning Kit (C115-01) were obtained from Vazyme (P.R. China.). Inhibitors including amlodipine (HY-B0317), Go6983 (HY-13689), AZ628 (HY-11004), and U0126 (HY-12031) were purchased from MedChemExpress (USA). The TSA inhibitor was purchased from Sigma (V900931, USA). pMT-ERK1 (12656), pcDNA-ERK2 (8974), and pcDNA-SOX9 (62972) were supplied by Addgene (USA). Fluo-4 AM (S1060), Nuclear and Cytoplasmic Protein Extraction Kit (P0028), and antibodies against β-actin (AF0003) and Lamin B1 (AF1408) were supplied by Beyotime (P.R. China.). Antibodies against total c-Raf (9422), phospho-c-Raf (9427), total MEK1/2 (9126), phospho-MEK1/2 (3958), total ERK1/2 (4695), phospho-ERK1/2 (4370), acetylated lysine (9681), GCN5L2 (3305), and IgG (5127) were provided by Cell Signaling Technology (USA). Antibodies against SOX9 (sc-166505 or ab3697), Cyclin D1 (ab16663), KAT7 (ab70183), PCAF (ab12188), total PKC-α (ab32376), phospho-PKC-α (ab32502), and phospho-(Ser/Thr) (ab17464) were purchased from Santa Cruz (USA) or Abcam (USA). Antibodies against phospho-SOX9 (BS4875) or HA (66006-2-Ig) were obtained from Bioworld (USA) or Proteintech (USA). A chromatin immunoprecipitation (ChIP) assay kit was purchased from Millipore (Bedford, MA, USA). The Dual-Luciferase^®^ Reporter Assay System (E1910) was obtained from Promega (USA).

### Cell culture, inhibitor pretreatment, and sublytic C5b‐9 stimulation

Rat GMCs were cultured in Minimum Essential Medium (MEM) containing 10% fetal bovine serum (FBS). The concentrations of Thy‐1 Ab and complement used in the study were 5% Thy‐1 Ab and 4% NHS (to form sublytic C5b‐9), as reported^28,29^. GMCs were divided into experimental groups and treated as follows: (a) MEM, (b) 5% Thy‐1 Ab, (c) 5% Thy‐1 Ab + 4% HIS, (d) 5% Thy‐1 Ab + 4% C6DS, (e) 5% Thy‐1 Ab + 4% C6DS + C6, or (f) 5% Thy‐1 Ab + 4% NHS. In the experiments, some GMCs were pretreated with inhibitors including amlodipine (10 μM), Go6983 (10 μM), AZ628 (10 μM), and U0126 (10 μM) for 30 min, followed by sublytic C5b-9 stimulation.

### CCK-8 assay

Rat GMCs at 0, 12, 24, and 36 h after various treatments were incubated with CCK-8 detection kits. The formazan product was visualized at an absorbance of 590 nm.

### EdU incorporation assay

Rat GMCs were cultured in MEM with 10% FBS. After 24 h, the cells were transfected with different plasmids or deprived of FBS for another 24 h. Next, the GMCs were stimulated with sublytic C5b-9 or other treatments for the indicated times. For the EdU assay, GMCs were incubated with 10 µM EdU for the last 6 h. Then, EdU staining and Hoechst 33342 staining were performed with Cell-Light™ EdU Apollo^®^567 In Vitro Imaging Kits. The ratios of EdU-positive cells to total cells (Hoechst 33342-positive cells) were calculated.

### Detection of intracellular calcium

The intracellular calcium level was measured with Fluo-4 AM according to the manufacturer’s instructions. In detail, GMCs treated with sublytic C5b-9 were incubated with Fluo-4 AM (final concentration of 2 μM) for 30 min in PBS at 37 °C, washed three times and incubated for an additional 10 min in the absence of Fluo-4 AM to complete the de-esterification process of the dye. The mean fluorescence intensity for each cell was calculated by ImageJ.

### Rat Thy-1N induction and experimental design

First, normal SD rats were injected with Thy‐1 Ab (0.75 ml/100 g, i.v.) to induce Thy‐1N. Normal rabbit serum (NRS) was injected (0.75 ml/100 g, i.v.) as a control. The rat renal cortex was obtained by sacrifice at different times and examined with immunoblot (IB) and coimmunoprecipitation (co-IP) assays.

Next, to confirm the roles of ERK1/2, SOX9, and Cyclin D1 in GMC proliferation in Thy-1N rats, male SD rats were divided into the following six groups (*n* = 5 at each time point/group): (1) NRS; (2) Thy-1N; (3) LV-shCTR + Thy-1N; (4) LV-shERK1/2 + Thy-1N; (5) LV-shSOX9 + Thy-1N; and (6) LV-shCyclin D1 + Thy-1N. Then, rat renal artery perfusion was performed with the corresponding LV-shRNA (2 × 10^7^ TU/ml LV-shRNA) for 4 days followed by NRS or Thy‐1 Ab (1 ml/100 g, i.v.) using the same method^30^. Rat renal cortex samples were collected after sacrifice at 3 h and on day 7 after treatment. The efficiency of gene silencing and the expression of indicated proteins were evaluated by IB, and SOX9 phosphorylation, acetylation and interaction with p-ERK1/2 were determined by co-IP. In addition, changes in the proliferation or lesions of renal tissues were determined by light microscopy (LM), electron microscopy (EM) and urine protein concentrations depicted as follows.

### MsPGN patient renal sample collection

Renal specimens were collected from patients who underwent kidney biopsy at the First Affiliated Hospital of Nanjing Medical University from 2015 to 2020, and 20 patients with an MsPGN diagnosis were enrolled. All renal specimens contained at least eight glomeruli available for scoring. Moreover, renal adjacent tissues taken from the unaffected pole of kidneys removed from renal cell carcinoma patients at the same hospital were collected as normal controls (*n* = 22). All patients and controls signed an informed consent document. The procedures involving the use of human tissues were approved by the Ethics Committee of Nanjing Medical University.

### RT-PCR and qRT-PCR

Total RNA was extracted using RNAiso Plus, and cDNA was synthesized using reverse transcriptase by HiScript III RT SuperMix for qPCR (+gDNA wiper). Reverse transcription-polymerase chain reaction (RT-PCR) and quantitative real-time PCR (qRT-PCR) were carried out with 2× Taq Master Mix (Dye Plus) on a SimpliAmp System (Thermo Fisher) and with SYBR Green Supermix on an ABI StepOne Plus System. For qRT-PCR, relative gene expression was calculated by the 2-^ΔΔct^ method. The primers are listed in Table S1.

### IB and co-IP assays

GMCs and renal tissues were lysed with RIPA or NE-PER lysis buffer containing a phosphatase inhibitor. Equal amounts (40 μg/lane) of protein were subjected to SDS-PAGE. IB analysis was performed^28^. β-actin was used as an internal control for protein loading, and the relative protein level in each group was calculated by comparison to that in the control group.

Co‐IP was performed to enrich the target protein complex^31–33^. Briefly, a total of 400 μg of extracts prepared from GMCs, cell nuclear, and cytoplasmic compartments or renal tissues were mixed with 40 μl Protein A/G-Sepharose Beads in co-IP assay buffer, incubated for 2 h and centrifuged for 2 min. The recovered supernatant was incubated with the corresponding Abs (2 g, IgG isotype as a control) at 4 °C for 12 h. Then, 40 ml of Protein A/G-Sepharose Beads were added, and the incubation was continued for 2 h. Protein A/G-precipitated protein complexes were recovered by centrifugation, and the harvested beads were resuspended in 50 μl of 2× SDS-PAGE sample buffer and boiled for 5 min. The samples were analyzed by IB. A 40 mg aliquot of whole-cell extract (WCE) was used as an input control.

### DNA constructs

pMT-ERK1, pcDNA-ERK2, and pcDNA-SOX9 (HA-tagged) plasmids were obtained from Addgene. pcDNA-Cyclin D1 was constructed by inserting the ORF of rat Cyclin D1 cDNA (NM_171992) into the pcDNA vector. Cyclin D1 cDNA was extracted from GMCs and then amplified by PCR. The PCR products, together with the pcDNA3.1 vector, were digested and ligated by the ClonExpress Ultra One Step Cloning Kit^34^. pcDNA-ERK1 and pcDNA-SOX9 (S64A/E, S149A/E, and S181A/E) were subcloned from pMT-ERK1 and wild-type (WT) pcDNA-SOX9 by General Biosystems. The primers used for the pcDNA-Cyclin D1 construct are listed in Table S1. To generate a constitutively activated rat ERK1/2 plasmid, the arginine 85 residue on rat ERK1, which amounts to the arginine 84 residue on human ERK1, was mutated into serine (R85S), and the arginine 65 residue on rat ERK2, which amounts to the arginine 67 residue on human ERK2, was mutated into serine (R65S)^35,36^. The pcDNA-ERK1 (R85S) and pcDNA-ERK2 (R65S) plasmids were directly obtained by gene synthesis.

Full-length pGL3-Cyclin D1 (FL) was constructed by inserting the 1.173-kb Cyclin D1 promoter (−1042 to +131 nt) into the pGL3-basic vector. To determine the minimal Cyclin D1 promoter sequence required for constitutive and inducible activity, we constructed promoter deletion fragments by PCR and cloned them into the same reporter vector: −985 to +130 nt and −582 to +130 nt. The specific primers used for FL and different deletion fragments are listed in Table S1.

To silence ERK1/2 and SOX9, different shRNA sequences against the mRNAs of ERK1, (NM_017347), ERK2 (NM_053842), SOX9 (NM_080403), and Cyclin D1 (NM_171992) were designed. The plasmids of shERK1, shERK2, shSOX9, and shCyclin D1 were constructed in the pGPU6/GFP vector, and the most effective shRNA was chosen. Additionally, shCTR was produced as a negative control. The sequences are listed in Table S2.

### Lentiviral shRNA packing

Lentiviral (LV)‐shERK1/2, LV‐shSOX9, LV-shCyclin D1, and LV-shCTR were provided by GenePharma (Shanghai, China). The oligonucleotide sequences of shCTR and of the shRNAs designed to silence ERK1/2, SOX9, and Cyclin D1 were the same as the sequences used in vitro.

### Cellular transfection

GMCs were transfected with the corresponding plasmids using the Neon™ Transfection System. A total of 4 × 10^5^ cells were resuspended in 100 μl resuspension buffer, including 3 μg plasmids, and electroporated at 1600 V (20 ms, 1 time). The cells were then transferred to a six-well plate.

### Luciferase reporter assay

The activities of the FL and deletion mutant Cyclin D1 promotors after sublytic C5b-9 stimulation or ERK1/2 and SOX9 overexpression were detected by luciferase reporter assays as reported previously^37^.

### ChIP-PCR

A ChIP assay was performed by using anti-SOX9 or IgG^37^. Three regions in the Cyclin D1 promoter (−668 to −414 nt, −562 to −145 nt, and −238 to −188 nt) were amplified from the immunoprecipitated chromatin by PCR. The specific primers used for the ChIP-PCR analysis are listed in Table S1.

### Subcellular localization

Subcellular localization was carried out by an NE-PER Kit (Pierce, Rockland, IL) followed by standard IB or co-IP. GMCs were transfected with different vectors or stimulated with different treatments. The cells were then trypsinized and lysed following the NE-PER protocol for cellular fractionation.

### Mass spectrometry

Mass spectrometric (MS) analyses were performed at the Center of Hygienic Analysis and Detection of Nanjing Medical University. GMCs were transfected with HA-tagged pcDNA-SOX9 for 36 h and deprived of serum for an additional 12 h. The cells were stimulated with sublytic C5b-9 for 3 h. The anti-HA immunoprecipitates were enriched from GMCs, followed by analysis with a mass spectrometer.

### Renal histological examination under LM and EM

For LM, renal tissue section (4 μm) were stained with hematoxylin and eosin (H&E) on day 7 after Thy-1N nephritis induction, and 100 glomerular cross-sections from each rat were examined^28^. For EM, ultrathin tissue sections were stained with uranyl acetate and lead citrate, and ultrastructural changes were observed.

### Urine protein detection

Twenty-four-hour rat urine samples were collected from the sixth day to the seventh day after Thy‐1N induction. The concentrations of urinary protein (mg/24 h) of rats were measured using total protein UC FS (DiaSys Diagnostic Systems, Holzheim, Germany).

### Immunohistochemical staining

Renal tissue sections were incubated with antibodies against p-ERK1/2, p‐SOX9, SOX9, and Cyclin D1 and then incubated with HRP‐conjugated anti‐rabbit IgG or HRP‐conjugated Streptavidin. Quantitative analysis (DAB staining) was performed using ImagePro Plus to determine the numbers of glomeruli positive for p‐ERK1/2, p-SOX9, SOX9, and Cyclin D1 expression.

### Data analysis

Data were presented as the mean ± SD. Significant differences among groups were determined by one-way ANOVA, and comparisons between two groups were analyzed by the *t* test. *p* < 0.05 was considered statistically significant.

## Results

### Sublytic C5b-9 stimulation arouses the GMC proliferative response

To confirm that sublytic C5b-9 induces GMC proliferation, we assessed GMC proliferative capacity in the presence of sublytic C5b-9 by CCK-8 and EdU incorporation assays. The results showed that sublytic C5b-9 stimulation for 24 h and 36 h promoted GMC proliferation (Supplementary Fig. S1a, c). To ensure that proliferation was indeed initiated upon sublytic C5b-9 treatment, GMCs were treated with MEM, Thy-1 Ab, Thy-1 Ab + HIS, Thy-1 Ab + C6DS, Thy-1 Ab + C6DS + C6, and Thy‐1 Ab + 4% NHS (which induces sublytic C5b-9 complex formation) for 24 h. GMC proliferation was the result of sublytic C5b-9 assembly, similar to the result of the Thy-1 Ab + 4% NHS group (Supplementary Fig. S1b, d), indicating that the formation of the sublytic C5b-9 on GMCs truly induces GMC proliferation.

### ERK1/2 phosphorylation and SOX9 or Cyclin D1 expression are upregulated both in GMCs exposed to sublytic C5b-9 and in the renal tissues of Thy-1N rats

Next, we performed IB and RT-PCR to quantify the levels of the potential kinase (ERK1/2), transcription factor (SOX9), and effector protein (Cyclin D1) responsible for GMC proliferation^38–40^. As shown in Supplementary Fig. S1e–h and Supplementary Fig. S2, ERK1/2 phosphorylation (p-ERK1/2) and SOX9 or Cyclin D1 expression increased in a time-dependent manner in GMCs treated with sublytic C5b-9 (in vitro) and in the renal tissues of Thy-1N rats (in vivo). Among them, p-ERK1/2 peaked at 2 h (in vitro) and 3 h (in vivo) (Supplementary Fig. S1e, g). The mRNA levels of SOX9 and Cyclin D1 reached a peak at 2 h (SOX9, in vivo) and 3 h (Supplementary Fig. S2a, c), respectively, and their maximum protein levels were observed at 3 and 6 h (Supplementary Fig. S1e, g).

In addition, we not only found higher levels of p-ERK1/2, SOX9, and Cyclin D1 in GMCs treated with Thy-1 Ab + C6DS + C6 or sublytic C5b-9 than in cells treated with MEM, Thy-1 Ab, Thy-1 Ab + HIS, or Thy-1 Ab + C6DS (Supplementary Figs. S1f and S2b), but also found higher levels of these proteins in the renal tissues of Thy-1N rats than in those of control rats (Supplementary Figs. S1h and S2d), confirming that the expression levels of p-ERK1/2, SOX9, and Cyclin D1 were truly increased.

### Sublytic C5b-9-induced ERK1/2 phosphorylation is driven by the PKC-α-c-Raf-MEK1/2 axis activated by calcium influx

It has been reported that sublytic C5b-9 can trigger calcium influx to activate the downstream kinase PKC, which induces canonical ERK1/2 signal transduction by regulating its upstream kinase Raf^41,42^. In addition, it has been proven that rat GMCs harbor L-type calcium channels^43^. Additionally, our in vitro and in vivo data demonstrated that PKC-α phosphorylation on Thr638, a hallmark of calcium influx, was stronger in Thy-1N rats (data were in preparation for submission). Hence, it is worth exploring whether calcium-PKC-α initiates ERK1/2 signal transduction in GMCs upon sublytic C5b-9 treatment.

By using the intracellular calcium indicator Fluo-4 AM, we observed an increase in the endogenous calcium level of rat GMCs stimulated with sublytic C5b-9 for 40 min, and this effect was prevented by the L-type calcium channel blocker amlodipine (Supplementary Fig. S3a). To further analyze the upstream pathways responsible for ERK1/2 activation in sublytic C5b-9-treated GMCs, we utilized different inhibitors to disrupt the influx of calcium or the activation of PKC (Go6983), Raf (AZ628), or MEK1/2 (U0126). The results showed that inactivation of c-Raf and MEK1/2 (upstream kinases of ERK1/2) dephosphorylated ERK1/2. In addition, blockade of calcium influx or the downstream kinase PKC-α ameliorated the phosphorylation of c-Raf, MEK1/2, and downstream ERK1/2 (Supplementary Fig. S3b). These data suggested that sublytic C5b-9-induced ERK1/2 phosphorylation is driven, at least in part, by the PKC-α-c-Raf-MEK1/2 axis activated by calcium influx.

### Sublytic C5b-9-induced GMC proliferation is mediated by activated ERK1/2 and upregulated SOX9 or Cyclin D1

**Fig. 1:**
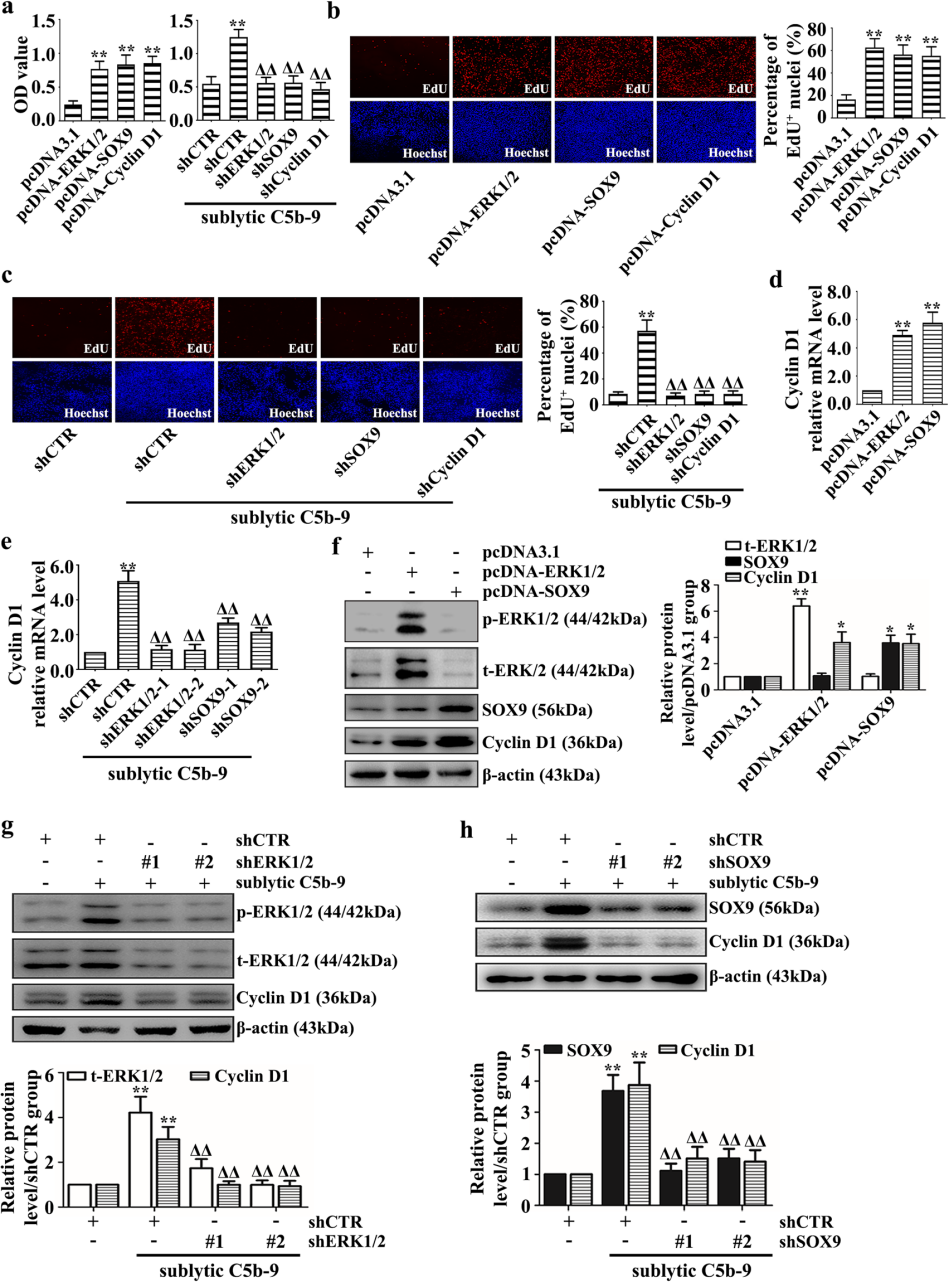
GMC proliferation and Cyclin D1 expression were regulated by ERK1/2, SOX9, or Cyclin D1 upon sublytic C5b-9 stimulation. **a**–**d** Rat GMCs overexpressing the pcDNA3.1-ERK1/2, pcDNA3.1-SOX9, and pcDNA3.1-Cyclin D1 plasmids were cultured for 48 h. **e**–**h** GMCs were stimulated with sublytic C5b-9 for 24 h or 3 h after transfection with the shERK1/2, shSOX9, or shCyclin D1 plasmids. The changes in cellular proliferation were tested by CCK-8 (**a**) or EdU incorporation assays (**b**, **c**). The mRNA and protein expression levels of Cyclin D1, t-ERK1/2, and SOX9 were assessed by qRT-PCR (**d**, **e**) and IB (**f**–**h**), respectively. *p < 0.05, **p < 0.01 versus the pcDNA3.1 group or the shCTR group; ^Δ^*p* < 0.05, ^ΔΔ^*p* < 0.01 versus the shCTR + sublytic C5b-9 group. Data were represented as the means ± SD (*n* = 5 in each group for the CCK‐8 and EdU incorporation assays, *n* = 3 in each group in the other experiments).

Our data showed that overexpression of ERK1/2, SOX9, or Cyclin D1 enhanced the GMC proliferative response, whereas knockdown of these proteins suppressed the increase in proliferative capacity induced by sublytic C5b-9 treatment (Fig. 1a–c). Moreover, to further confirm the role of ERK1/2 activation in GMC proliferation, we transfected constitutively activated rat ERK1/2 plasmids (including ERK1 (R85S) and ERK2 (R65S)) into GMCs^35,36^ or pretreated cells with U0126 (a MEK1/2 inhibitor) to dephosphorylate ERK1/2, followed by sublytic C5b-9 stimulation. As expected, constitutive activation of ERK1/2 dramatically promoted GMC proliferation (Supplementary Fig. S4a, b), whereas ERK1/2 dephosphorylation inhibited cell proliferation upon sublytic C5b-9 treatment (Supplementary Fig. S5a, b). These results suggest that ERK1/2 activation and SOX9 or Cyclin D1 upregulation play a role in GMC proliferation mediated by sublytic C5b-9.

### ERK1/2 and SOX9 boost Cyclin D1 expression in GMCs treated with sublytic C5b-9

Given that the change in p-ERK1/2 and SOX9 expression is approximately synchronous with or a little earlier than that of Cyclin D1 in GMCs exposed to sublytic C5b-9 and in the renal tissues of Thy-1N rats, we wondered whether sublytic C5b-9-elevated p-ERK1/2 and SOX9 are contributors to Cyclin D synthesis. To our surprise, GMCs overexpressing ERK1/2 or SOX9 exhibited increased Cyclin D1 production and silencing of these genes decreased Cyclin D1 expression in GMCs upon sublytic C5b-9 attack (Fig. 1d–h). Similarly, constitutively activating or inhibiting ERK1/2 activity also increased or reduced Cyclin D1 levels (Supplementary Figs. S4c and S5c,S5d), respectively, further indicating that sublytic C5b-9-induced Cyclin D1 upregulation may be mediated, to a large extent, by ERK1/2 activation and SOX9 induction.

### ERK1/2 facilitates the binding of SOX9 to the Cyclin D1 gene promoter in rat GMCs

**Fig. 2:**
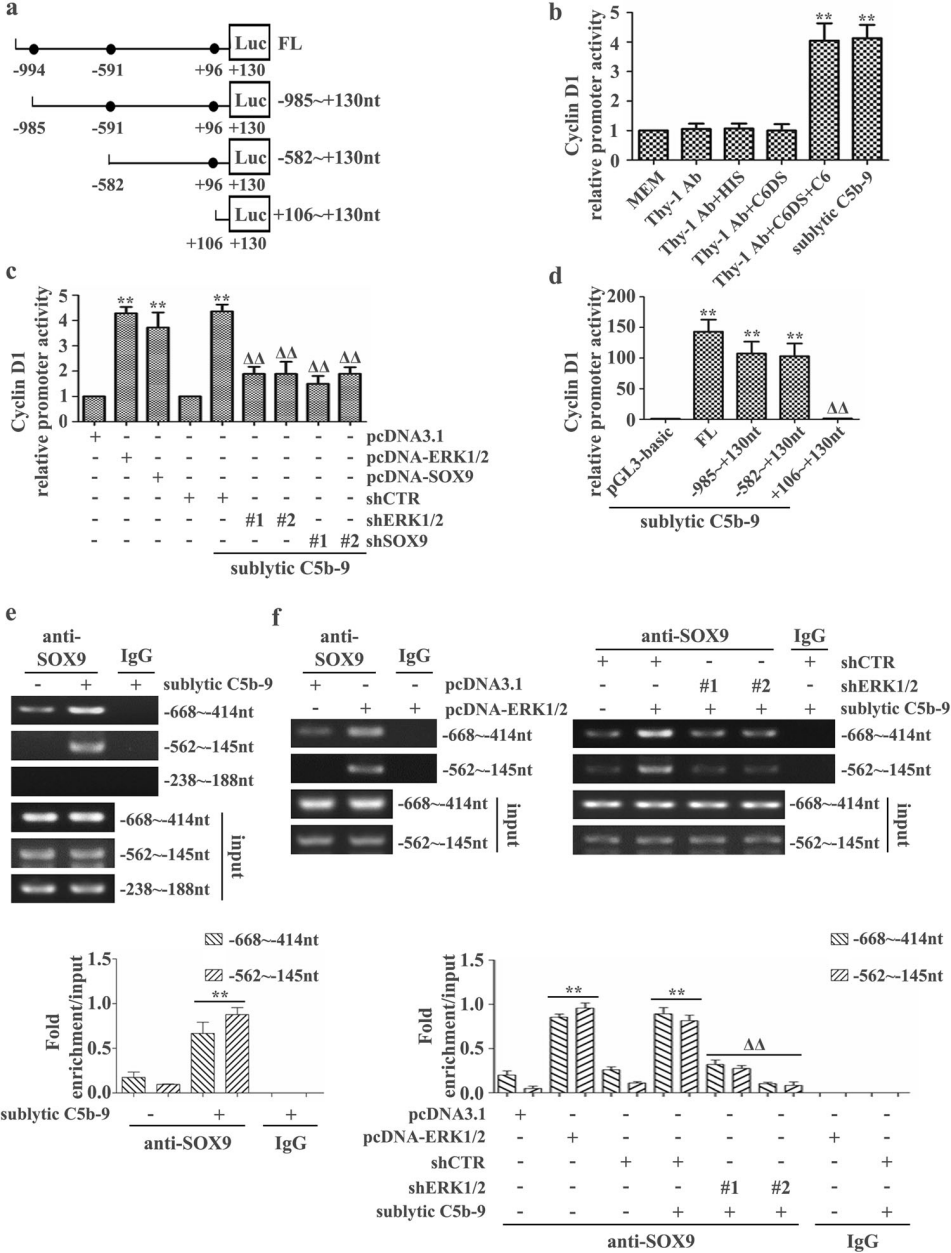
Cyclin D1 promoter activity and SOX9 binding elements in the Cyclin D1 promoter were affected by sublytic C5b-9 or the expression of SOX9 and ERK1/2. **a** Scheme showing full-length (FL) and the three indicated deletion mutants of the pGL-Cyclin D1 promoter according to JASPAR prediction. **b**–**f** Rat GMCs were cultured with various treatments or transfected with different plasmids with or without sublytic C5b-9 stimulation for 3 h. The activity of the Cyclin D1 promoter and the binding elements of SOX9 were measured by luciferase reporter assay (**b**–**d**) and ChIP-PCR (**e**, **f**). **p < 0.01 versus the MEM, Thy-1 Ab, Thy-1 Ab + HIS, or Thy-1 Ab + C6DS group or versus the pcDNA3.1, shCTR, or pGL3-basic + sublytic C5b-9 group; ^ΔΔ^*p* < 0.01 versus the shCTR + sublytic C5b-9, FL + sublytic C5b-9, -985 to +130 nt + sublytic C5b-9, or -582 to +130 nt + sublytic C5b-9 group. Data were represented as the means ± SD (*n* = 3 in each group in all experiments).

Since gene expression is modulated by diverse transcriptional mechanisms occurring at the promoter^44,45^, we asked whether sublytic C5b-9-upregulated SOX9 influences Cyclin D1 gene transcription. To identify the promoter region for Cyclin D1 transcription in response to SOX9 and sublytic C5b-9 exposure, we used JASPAR (http://jaspar.genereg.net/)^46^ to preliminarily predict the potential responsive elements on the rat Cyclin D1 promoter that are recognized by SOX9. The results showed that there were three potential SOX9 binding elements on the Cyclin D1 promoter, including −994 to −96 nt, −591 to −583 nt and +96 to +105 nt (Fig. 2a). Notably, sublytic C5b-9 stimulation enhanced Cyclin D1 promoter (FL) activity (Fig. 2b). Moreover, SOX9 overexpression or downregulation enhanced or reduced Cyclin D1 promoter activity (Fig. 2c), respectively, implying that sublytic C5b-9-upregulated SOX9 promotes Cyclin D1 gene transcription.

To identify SOX9 binding motif(s) on the Cyclin D1 promoter, we generated a series of deletion fragments of the Cyclin D1 promoter (−985 to +130, −582 to +130, and +106 to +130 nt) based on the prediction results. The luciferase reporter assay demonstrated that there was a marked decrease in promoter activity in GMCs transfected with the Cyclin D1 promoter (+106 to +130 nt) (Fig. 2d), indicating that SOX9 binding motifs may be located between −582 and +106 nt. Furthermore, a sharp increase or decrease in Cyclin D1-FL promoter activity was observed in GMCs after pcDNA-ERK1/2 or shERK1/2 plasmid transfection (Fig. 2c). Moreover, similar results were obtained in GMCs overexpressing constitutively activated rat ERK1/2 plasmids or pretreated with U0126 followed by sublytic C5b-9 stimulation (Supplementary Figs. S4d and S5e), hinting that sublytic C5b-9-enhanced Cyclin D1 transcription can be manipulated by SOX9 and ERK1/2.

To further ascertain the SOX9 binding motif on the Cyclin D1 promoter and to verify whether sublytic C5b-9-induced ERK1/2 activation contributes to SOX9 binding to the Cyclin D1 promoter, a ChIP-PCR assay was performed. The results showed that sublytic C5b-9 treatment, ERK1/2 overexpression or activation promoted the direct occupation of SOX9 at the promoter regions of −668 to 414 nt and −562 to −145 nt instead of −238 to +188 nt, whereas ERK1/2 silencing or inactivation limited SOX9 recruitment to these regions of the Cyclin D1 promoter in the presence of sublytic C5b-9 (Fig. 2e, f and Supplementary Figs. S4e, S5f), indicating that ERK1/2 activation facilitates SOX9 occupation at the −582 to −238 nt region of the Cyclin D1 promoter, causing its transcription.

### Sublytic C5b-9 stimulation activates the ERK1/2-SOX9 axis to boost GMC proliferation and Cyclin D1 expression

**Fig. 3:**
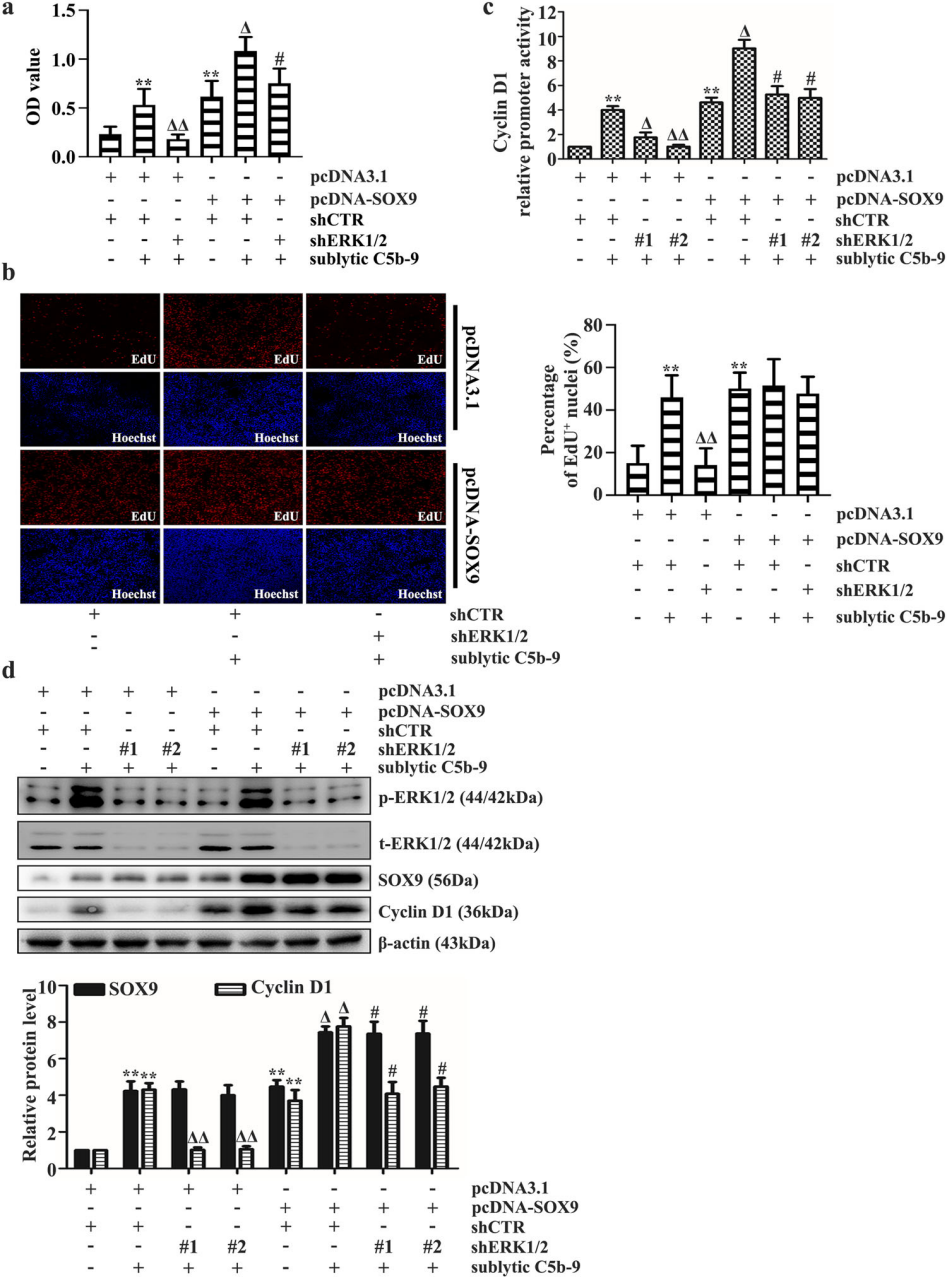
Role of the ERK1/2-SOX9 axis in sublytic C5b-9-exposed GMCs during cell proliferation and Cyclin D1 induction after shERK1/2 transfection. **a**–**d** Rat GMCs cotransfected with pcDNA-SOX9 and shERK1/2 plasmids were stimulated with sublytic C5b-9 for the indicated times. GMC proliferation was determined by CCK-8 (**a**) and EdU incorporation assays (**b**). Cyclin D1 promoter activity and protein level were analyzed by luciferase reporter assay (**c**) and IB (**d**), respectively. **p < 0.01 versus the pcDNA3.1 + shCTR group; ^Δ^*p* < 0.05, ^ΔΔ^*p* < 0.01 versus the pcDNA3.1 + shCTR + sublytic C5b-9 group; ^#^*p* < 0.05 or ^##^*p* < 0.01 versus the pcDNA3.1 + shERK1/2 + sublytic C5b-9 group. Data were represented as the means ± SD (*n* = 5 in each group for the CCK‐8 and EdU incorporation assays, *n* = 3 in each group in the other experiments).

To verify whether sublytic C5b-9-activated ERK1/2 leads to cell proliferation and whether Cyclin D1 expression is SOX9 dependent, GMCs were cotransfected with shERK1/2 and pcDNA-SOX9 plasmids or transfected with pcDNA-SOX9 followed by U0126 treatment and sublytic C5b-9 attack. The data showed that GMCs transfected with pcDNA-SOX9 exhibited a higher proliferative capacity (Fig. 3a, b and Supplementary Fig. S6a, b) and stronger Cyclin D1 promoter activity (Fig. 3c and Supplementary Fig. S6c) and expression (Fig. 3d and Supplementary Fig. S6d), proving that SOX9 overexpression can partially induce cell proliferation and Cyclin D1 synthesis in sublytic C5b-9-treated GMCs after ERK1/2 gene silencing or activity blockade. Unexpectedly, we did not observe a sharp increase in SOX9 abundance provoked by ERK1/2 overexpression (Fig. 1f) or a decline in SOX9 protein levels in sublytic C5b-9-treated GMCs after ERK1/2 knockdown or blockade (Fig. 3d and Supplementary Fig. S6d), suggesting that ERK1/2 may influence SOX9 activity rather than its abundance. Collectively, these findings suggest that ERK1/2 may be an upstream regulator of SOX9 and that the ERK1/2-SOX9 axis elevates GMC proliferation and Cyclin D1 expression in cells stimulated by sublytic C5b-9.

### SOX9 phosphorylation and interaction with p-ERK1/2 are induced in GMCs attacked by sublytic C5b-9 and in the renal tissues of Thy-1N rats

**Fig. 4:**
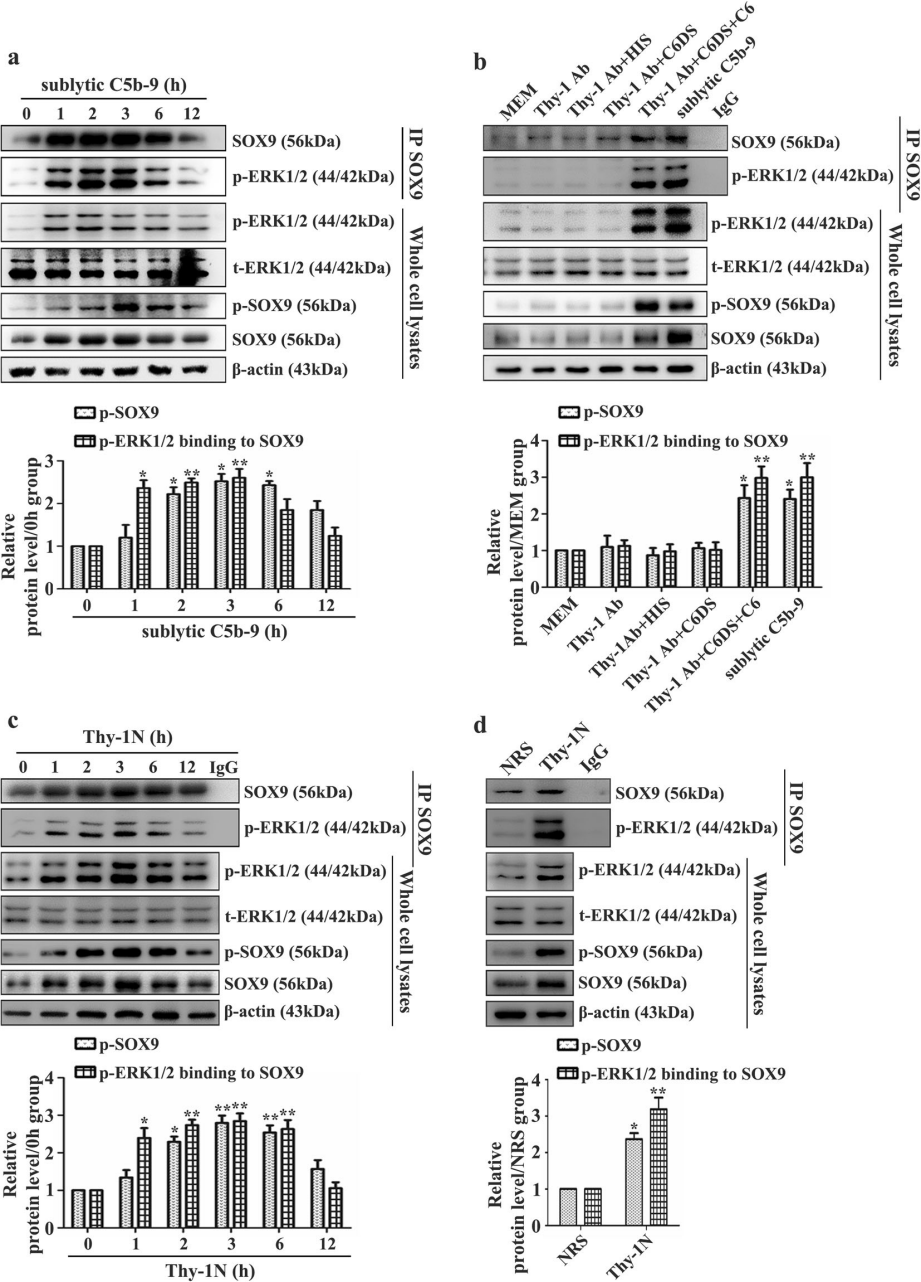
Phosphorylation level of SOX9 and its interaction with p-ERK1/2 in GMCs following sublytic C5b-9 exposure and in renal tissues of Thy-1N mice. **a** The protein levels of t-ERK1/2, p-SOX9, and SOX9 and the interaction between SOX9 and p-ERK1/2 in sublytic C5b-9-treated rat GMCs at different time points. **b** The protein levels of t-ERK1/2, p-SOX9, and SOX9 and the interaction between SOX9 and p-ERK1/2 in GMCs at 3 h with various treatments. **c** The protein levels of t-ERK1/2, p-SOX9, and SOX9 and the interaction between SOX9 and p-ERK1/2 in renal tissue of Thy-1N rats at different time points. **d** The protein levels of t-ERK1/2, p-SOX9, and SOX9 and the interaction between SOX9 and p-ERK1/2 at 3 h in the renal tissues of Thy-1N and NRS control rats. *p < 0.05, **p < 0.01 versus the 0 h, MEM, Thy-1 Ab, Thy-1 Ab + HIS, Thy-1 Ab + C6DS, or NRS group. Data were represented as the means ± SD (*n* = 3 in vitro, *n* = 5 in vivo at each time point or in each group).

In this experiment, we used an antibody against the rat Ser181 residue of SOX9 (a known phosphorylation site) to evaluate its activity. Then, we detected the interaction between SOX9 and p-ERK1/2 following sublytic C5b-9 attack (in vitro) and in the renal tissues of Thy-1N rats (in vivo). IB and co-IP data showed that SOX9 phosphorylation at Ser181 and its association with p-ERK1/2 were all increased, with the maximum observed at 3 h (Fig. 4a, c). Moreover, SOX9 phosphorylation (Ser181) and binding with p-ERK1/2 were also enhanced both in vitro and in vivo (Fig. 4b, d), indicating that during Thy-1N, sublytic C5b-9-activated ERK1/2 phosphorylates SOX9 in rat GMCs.

### Sublytic C5b-9-activated ERK1/2 mediates SOX9 phosphorylation and acetylation upon binding to SOX9 in the GMC nucleus

**Fig. 5:**
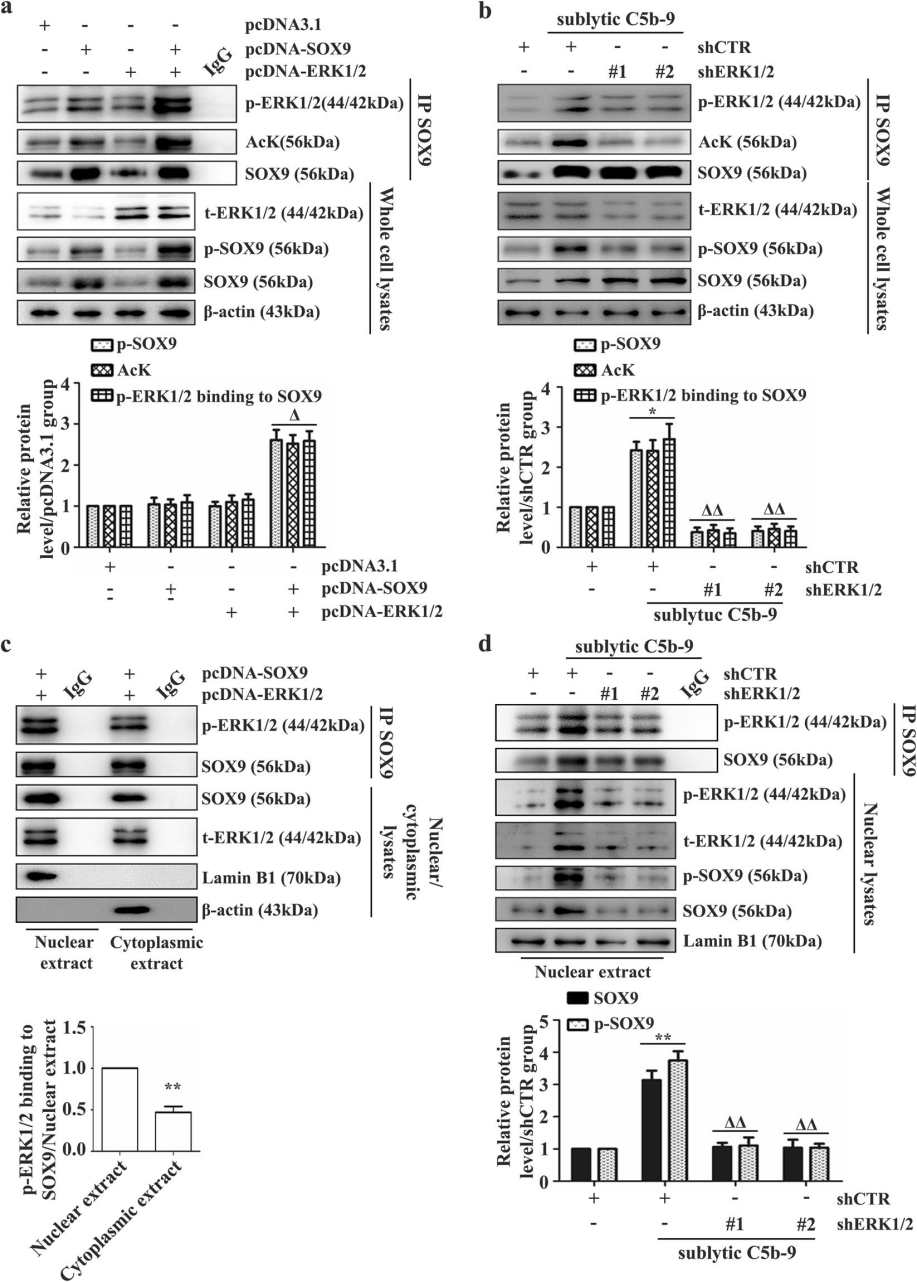
Contribution of ERK1/2 activation to SOX9 phosphorylation, acetylation and nuclear expression. **a**, **c** Rat GMCs overexpressing the pcDNA3.1-SOX9 and pcDNA3.1-ERK1/2 plasmids were cultured for 48 h. **b**, **d** In addition, GMCs were stimulated with sublytic C5b-9 for 3 h after transfection with the shERK1/2 plasmid. The interaction of SOX9 with p-ERK1/2 and the SOX9 acetylation level were measured by co-IP, and the SOX9 phosphorylation level was tested by IB (**a**, **b**). GMC cytoplasmic and nuclear proteins were isolated, and SOX9 was immunoprecipitated from the two fractions followed by IB with SOX9 and p-ERK1/2 antibodies (**c**, **d**). *p < 0.05, **p < 0.01 versus the shCTR group or nuclear extract; ^Δ^*p* < 0.05, ^ΔΔ^*p* < 0.01 versus the pcDNA-SOX9, pcDNA-ERK1/2, or shCTR + sublytic C5b-9 group. Data were represented as the means ± SD (*n* = 3 in each group in all experiments).

We transfected pcDNA-ERK1/2 into GMCs, and then the levels of SOX9 phosphorylation and binding to p-ERK1/2 were measured by IB and co-IP. As expected, GMCs harboring exogenous ERK1/2 and SOX9 exhibited an intense increase in SOX9 phosphorylation and physical interaction between SOX9 and p-ERK1/2 (Fig. 5a), and these effects were weakened when ERK1/2 expression or activity induced by sublytic C5b-9 was blocked (Fig. 5b and Supplementary Fig. S7a).

Given that SOX9 phosphorylation at Ser181 can determine its nuclear localization^47,48^, we checked the interaction of SOX9 with p-ERK1/2 in both the nuclear and cytoplasmic compartments. Co-IP showed that SOX9 bound higher amounts of p-ERK1/2 in the GMC nucleus (Fig. 5c). Further co-IP and IB showed that silencing the ERK1/2 gene or blocking its activity not only reduced SOX9 phosphorylation provoked by sublytic C5b-9 but also decreased its protein level in the cell nucleus (Fig. 5d and Supplementary Fig. S7b), suggesting that sublytic C5b-9-activated ERK1/2 mediates SOX9 phosphorylation and its interaction with p-ERK1/2 in the GMC nucleus.

**Fig. 6:**
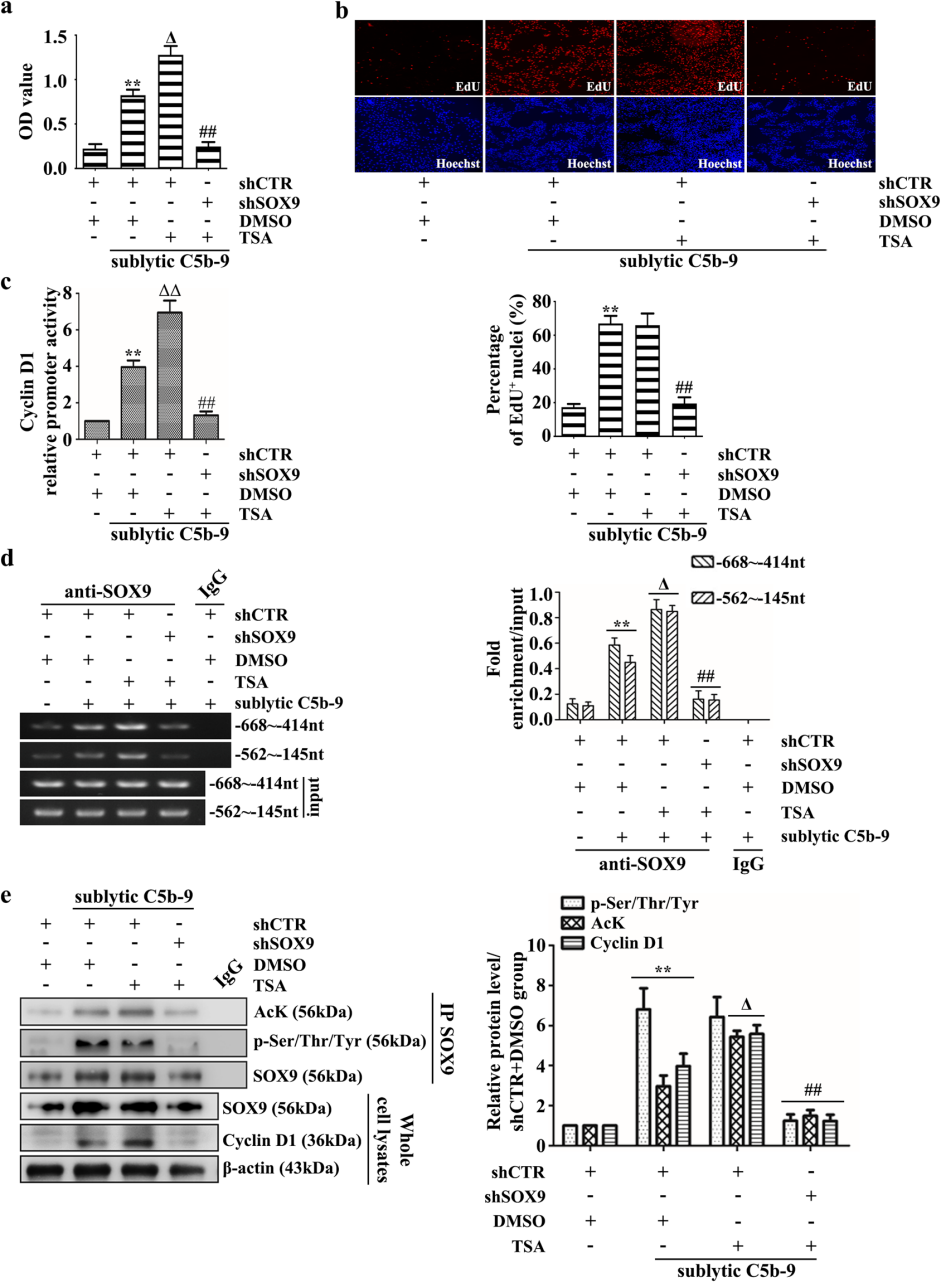
The dependency of acetylated SOX9 on SOX9 phosphorylation for Cyclin D1 gene induction and GMC proliferation in response to sublytic C5b-9. Rat GMCs pretransfected with shSOX9 plasmid were treated with TSA (20 μM) for 30 min, followed by sublytic C5b-9 stimulation for 24 h or 3 h. GMC proliferation was determined by CCK-8 (**a**) and EdU incorporation assays (**b**). Cyclin D1 promoter activity and occupation by SOX9 were analyzed by luciferase reporter assay (**c**) and ChIP-PCR (**d**). The levels of SOX9 acetylation and phosphorylation and the protein expression of Cyclin D1 were determined by co-IP and IB (**e**). **p < 0.01 versus the shCTR + DMSO group; ^Δ^*p* < 0.05 or ^ΔΔ^*p* < 0.01 versus the shCTR + DMSO + sublytic C5b-9 group; ^##^*p* < 0.01 versus the shCTR + TSA + sublytic C5b-9 group. Data were represented as the means ± SD (*n* = 5 in each group for CCK‐8 assays, *n* = 3 in each group in the other experiments).

Reportedly, SOX9 acetylation can also affect its transcriptional activity^49^, and our previous studies showed that sublytic C5b-9 enhanced SOX9 acetylation (data not shown), with an increase in HATs, i.e., KAT7, PCAF, and GCN5^33^. In addition, we found that HDAC inhibitor trichostatin A (TSA)-mediated SOX9 hyperacetylation in GMCs could further increase cell proliferation induced by sublytic C5b-9 (Fig. 6a, b, e). Next, we also evaluated the influence of SOX9 acetylation on Cyclin D1 transcription and expression, and the data showed that Cyclin D1 promoter activity and expression, including SOX9 occupation at the Cyclin D1 promoter, were significantly enhanced when cells were treated with TSA and sublytic C5b-9 (Fig. 6c–e). These data suggest that SOX9 acetylation indeed improved its transcriptional activity to induce Cyclin D1 gene expression for GMC proliferation. Moreover, to our surprise, overexpression or knockdown of the ERK1/2 gene greatly enhanced or repressed SOX9 acetylation upon sublytic C5b-9 treatment (Fig. 5a, b), indicating that sublytic C5b-9-mediated ERK1/2 phosphorylation may indirectly promote SOX9 acetylation. More importantly, blockade of ERK1/2 activity not only reduced SOX9 acetylation but also prevented its combination with KAT7, PCAF, and GCN5 (Supplementary Fig. S7a). Furthermore, ERK1/2 overexpression also increased GCN5 protein levels (Supplementary Fig. S8a), and this effect was prevented by ERK1/2 gene knockdown or activity blockade (Supplementary Fig. S8b, c).

### SOX9 phosphorylation at Ser64 and Ser181 is required for its nuclear expression, phosphorylation, and acetylation as well as Cyclin D1 induction and GMC proliferation

**Fig. 7:**
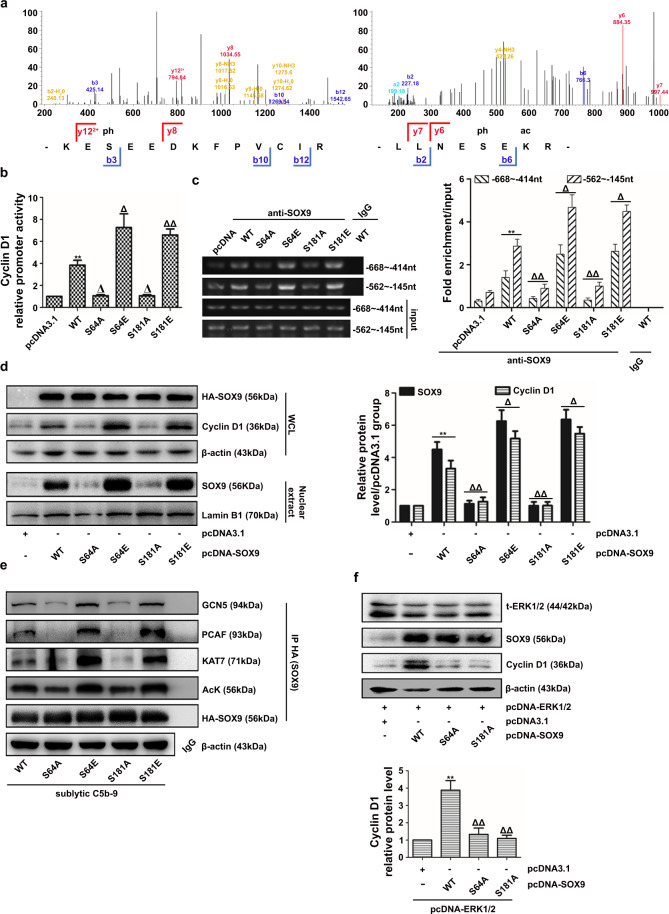
Identification of SOX9 phosphorylation sites modified by ERK1/2 and their influence on Cyclin D1 expression, SOX9 nuclear location and acetylation. **a**–**e** Rat GMCs were transfected with HA-tagged pcDNA-SOX9 wild-type (WT) or different phospho-mutant pcDNA-SOX9 plasmids (S64A/E or S181A/E) in the presence or absence of sublytic C5b-9. HA-SOX9 protein was purified, and the SOX9 phosphorylation sites were identified by mass spectrometry (MS, **a**). Cyclin D1 promoter activity and promoter occupation were determined by luciferase reporter (**b**) and ChIP-PCR assays (**c**). Cyclin D1 abundance and nuclear SOX9 levels were tested by IB (**d**). SOX9 acetylation and its association with KAT7, PCAF, and GCN5 were measured by co-IP assays (**e**). **f** Rat GMCs overexpressing the ERK1/2 gene were transfected with pcDNA-SOX9 WT or different phospho-mutant pcDNA-SOX9 plasmids for 48 h. The Cyclin D1 protein level was measured by IB. **p < 0.01 versus the pcDNA3.1 or pcDNA-ERK1/2 + pcDNA3.1 group; ^Δ^*p* < 0.05, ^ΔΔ^*p* < 0.01 versus the WT or pcDNA-ERK1/2 + WT group. Data were represented as the means ± SD (*n* = 3 in each group in all experiments).

To precisely identify other SOX9 phosphorylation residues that might be mediated by sublytic C5b-9-activated ERK1/2, GMCs were transfected with the HA-tagged pcDNA-SOX9 vector, followed by sublytic C5b-9 stimulation. Then, the HA tag was enriched and collected for MS analysis. The MS data exhibited two phosphorylation sites on SOX9 serine residues, namely, Ser64 and Ser149 (Fig. 7a). Unexpectedly, the canonical Ser181 residue was not detected. We considered that the signal of phosphorylation on Ser181 of SOX9 did not reach the threshold set by MS detection.

To explore the role of SOX9 phosphorylation at these residues in GMC proliferation, Cyclin D1 synthesis and SOX9 nuclear expression, we generated phosphorylation-inactivated or phosphorylation-activated SOX9 mutants by replacing each serine with alanine or glutamic acid (S64A/E, S149A/E, and S181A/E, Supplementary Fig. S9a). It is worth noting that GMCs expressing the SOX9 S64A and S181A mutants displayed a reduced proliferative ability (Supplementary Fig. S9b, S9c). In contrast, SOX9 hyperphosphorylation at Ser64 (S64E) and Ser181 (S181E) increased the GMC number, although the percentages of GMCs in S phase remained unaltered (Supplementary Fig. S9b). Additionally, transfection with SOX9 bearing phosphorylation-inactivating or phosphorylation-activating mutations at the two sites notably downregulated or upregulated Cyclin D1 promoter activity, occupation and its expression as well as SOX9 abundance in the GMC nucleus (Fig. 7b–d). Surprisingly, hypophosphorylation of SOX9 at either Ser64 or S181 significantly ameliorated the interactions between SOX9 and KAT7, PCAF, and GCN5, resulting in a decline in SOX9 acetylation, and vice versa (Fig. 7e). These results indicate that SOX9 phosphorylation at Ser64 and Ser181 can facilitate its nuclear expression and acetylation, finally causing increased Cyclin D1 transcription that promotes GMC proliferation.

Furthermore, we cotransfected the ERK1/2 overexpression plasmid with different phospho-inactivated SOX9 mutant or WT plasmids into GMCs. The IB data showed that inadequate phosphorylation of SOX9 at Ser64 and Ser181 markedly decreased Cyclin D1 protein levels in GMCs upon ERK1/2 overexpression (Fig. 7f), revealing that SOX9 phosphorylation at Ser64 and Ser181 is ERK1/2-dependent.

Moreover, to investigate whether SOX9 acetylation also influences its phosphorylation, GMCs were pretreated with TSA, followed by sublytic C5b-9 treatment. As mentioned above, although the co-IP results confirmed that sublytic C5b-9-induced SOX9 acetylation was enhanced in the presence of TSA, its phosphorylation remained at the same level (Fig. 6e), indicating that SOX9 acetylation does not affect its phosphorylation and that SOX9 acetylation is a downstream event of its phosphorylation in response to sublytic C5b-9.

### Knockdown of the renal ERK1/2, SOX9, and Cyclin D1 genes in vivo represses GMC proliferation and urinary protein secretion in Thy-1N rats

**Fig. 8:**
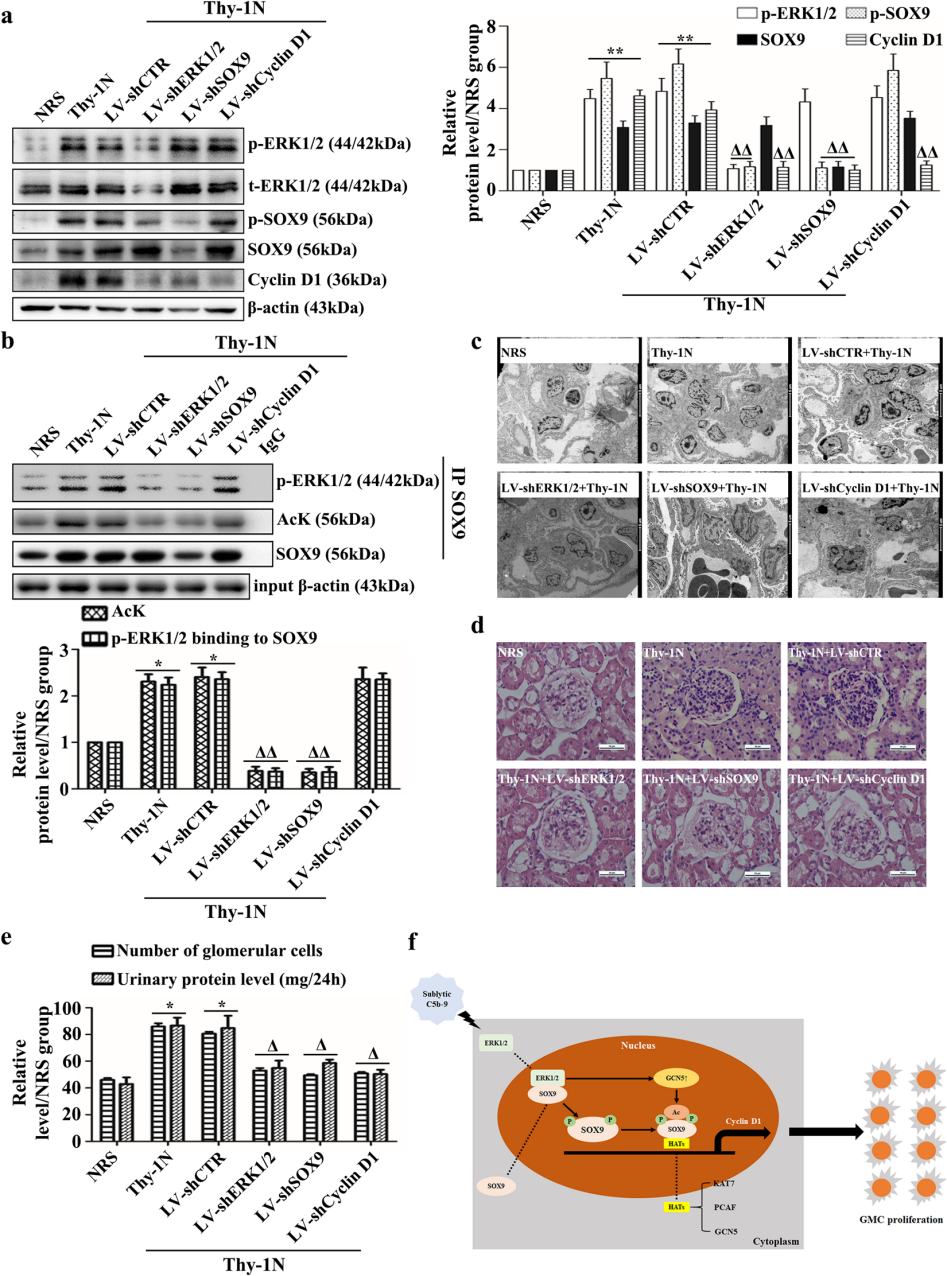
Effects of ERK1/2, SOX9 and Cyclin D1 gene knockdown on pathological changes and urinary protein secretion in Thy-1N rats. **a**, **b** The protein levels of ERK1/2 (t-ERK1/2 and p-ERK1/2), SOX9 (SOX9 and p-SOX9), and Cyclin D1 (**a**) as well as SOX9 acetylation and its interaction with p-ERK1/2 (**b**) were measured at 3 h in the renal tissues of Thy-1N from different groups as indicated. **c** Renal ultrastructural changes on day 7 were examined by EM. **d**, **e** The changes in glomerular cell number in each group on day 7 were examined by H&E staining under LM. **e** The total contents of urinary protein (mg/24 h) on day 7 were detected. *p < 0.05, **p < 0.01 versus the NRS group; ^Δ^*p* < 0.05, ^ΔΔ^*p* < 0.01 versus the LV-shCTR + Thy-1N group. Data were represented as the means ± SD (*n* = 5 in vivo in each group). **f** A putative scheme for the molecular regulation of GMC proliferation triggered by sublytic C5b-9. In response to extracellular sublytic C5b-9 attack, ERK1/2 is phosphorylated and enters the GMC nucleus to form a complex with SOX9, leading to SOX9 phosphorylation at the Ser64 and Ser181 residues. Phosphorylation of SOX9 at these sites not only induces SOX9 nuclear localization but also enhances its acetylation by promoting the interaction of SOX9 with HATs, including KAT7, PCAF, and GCN5. In addition, ERK1/2-upregulated GCN5 is also implicated in SOX9 acetylation. Nuclear phosphorylated or acetylated SOX9 can be recruited to the Cyclin D1 promoter regions, causing its gene induction and GMC proliferation.

To further determine whether ERK1/2, SOX9, or Cyclin D1 activation or upregulation can play a proliferative role, rat Thy-1N was established for renal gene knockdown via renal artery perfusion of LV-shRNAs^33^. The in vivo IB results showed that the protein levels of ERK1/2 (t-ERK1/2, p-ERK1/2), SOX9 (SOX9, p-SOX9), and Cyclin D1 decreased in the renal tissues of Thy-1N rats upon LV-shERK1/2, LV-shSOX9, or LV-shCyclin D1 pretreatment, respectively (Fig. 8a). In addition, LV-shERK1/2 pretreatment also decreased Cyclin D1 expression and SOX9 phosphorylation, acetylation, and association with p-ERK1/2 (Fig. 8a, b). Furthermore, Thy-1N rats pretreated with LV-shERK1/2, LV-shSOX9, or LV-shCyclin D1 exhibited suppressed GMC proliferation, ECM accumulation, and urinary protein production (Fig. 8c–e), proving that knockdown of the renal ERK1/2, SOX9, or Cyclin D1 gene indeed alleviates GMC proliferation and other lesions in Thy-1N rats.

### Phosphorylation or expression of ERK1/2, SOX9, and Cyclin D1 is elevated in renal tissues of MsPGN patients

We examined the expression of these proteins in MsPGN patients by immunohistochemical staining (IHC). Renal tissue sections displayed positive expression of p-ERK1/2, p-SOX9, SOX9, and Cyclin D1 (Supplementary Fig. S10a, S10b). Additionally, the expression of p-ERK1/2, p-SOX9, and SOX9 was positively correlated with that of Cyclin D1 (*r*^2^ = 0.7357, *p* < 0.01; *r*^2^ = 0.5425, *p* < 0.01; *r*^2^ = 0.2143, *p* = 0.03), and p-ERK1/2 also showed a positive correlation with p-SOX9 (*r*^2^ = 0.5110, *p* < 0.01) but not SOX9 (*r*^2^ = 0.1110, *p* = 0.1511) (Supplementary Fig. S10c). These results indicate that ERK1/2, SOX9, and Cyclin D1 expression may play a role in MsPGN.

## Discussion

Revelation of the mechanisms by which sublytic C5b-9 triggers GMC proliferation in rat Thy-1N is essential for understanding human MsPGN. Although our published work has demonstrated that PI3K-Akt signaling and the ERK5-MZF1-RGC‐32 axis are involved in GMC proliferation induced by sublytic C5b-9 attack, silencing of these genes did not completely ameliorate proliferation^9,11^, indicating that there might exist other mechanisms that can cause proliferative lesions in Thy-1N rats.

At the beginning of the experiments, we determined the time required for sublytic C5b-9 exposure to trigger GMC proliferation in vitro, and the data showed that sublytic C5b-9 markedly induced GMC proliferation at 24 and 36 h.

While investigating the signaling pathways responsible for GMC proliferation triggered by sublytic C5b-9, we considered ERK1/2 as a candidate for three reasons: (a) ERK1/2 is activated in GMCs in response to sublytic C5b-9^50^; (b) activation of ERK1/2 promotes cell proliferation in most situations^38,51^; and (c) our previous work suggested an proapoptotic role of ERK1/2 inhibition induced by sublytic C5b-9^50^. In this study, we first confirmed the enhancement of ERK1/2 phosphorylation both in GMCs subjected to sublytic C5b-9 attack (in vitro) and in the renal tissues of Thy-1N rats (in vivo), and proved that ERK1/2 had a pro-proliferative role. In addition, we not only preliminarily revealed the mechanism by which sublytic C5b-9 treatment of rat GMCs elevates calcium influx, resulting in PKC-α-c-Raf-MEK1/2 axis-dependent ERK1/2 activation, but also proved that the transcription factor SOX9 and the cell cycle regulator cyclin D1 were markedly increased both in vitro and in vivo.

Although SOX9 or Cyclin D1 can promote cell proliferation^20,52,53^, their effects on GMC proliferation in the Thy-1N rats have yet to be expounded. Our data showed that overexpression or activation of ERK1/2, SOX9, and Cyclin D1 boosted GMC proliferation. Considering that the increased expression of p-ERK1/2 and SOX9 is highly synchronous with that of Cyclin D1, together with the findings that ERK1/2 induces Cyclin D1 expression with pro-proliferative activity^54^ and SOX9 enhances cell proliferation by activating signals^55,56^, it is worth exploring whether Cyclin D1 levels are also manipulated by ERK1/2 and SOX9 in sublytic C5b-9-treated GMCs remains unclear. In this study, we observed Cyclin D1 upregulation in GMCs via ERK1/2 and SOX9 overexpression or constitutive activation. Additionally, reduced Cyclin D1 expression was observed in GMCs transfected with shERK1/2 and shSOX9 plasmids or pretreated with U0126, followed by sublytic C5b-9 stimulation. These data imply that upregulation or activation of ERK1/2 and SOX9 promotes GMC proliferation by enhancing Cyclin D1 expression.

SOX9 is required for the recognition of the DNA motif C(A/T)TTG(A/T)(A/T) in the promoter regions of target genes^57,58^. Although the rat Cyclin D1 promoter harbors potential SOX9 binding sites, the direct effect and molecular mechanism of SOX9 in Cyclin D1 gene induction were unknown. Additionally, several studies have reported that ERK1/2 is responsible for SOX9 upregulation^59,60^, suggesting that Cyclin D1 expression mediated by ERK1/2 might be SOX9 dependent. In this experiment, we not only identified novel SOX9 binding elements on the Cyclin D1 promoter (−582 to −238 nt) but also revealed for the first time that ERK1/2 activation augmented SOX9 recruitment to the Cyclin D1 promoter. Nevertheless, while SOX9 upregulation was found to reduce GMC proliferation and Cyclin D1 expression, its endogenous or exogenous abundance was not affected by ERK1/2, which was inconsistent with the report that preadipocyte factor 1 or epidermal growth factor receptor-induced ERK1/2 activation enhanced SOX9 protein levels^59,60^, indicating that sublytic C5b-9-activated ERK1/2 may alter the function of SOX9 in addition to its protein level. Recently, our group also found activation or upregulation of other transcription factors, e.g., NF-κB and KLF5 (data not shown), in GMCs stimulated with sublytic C5b-9 (in vitro) and in the early stage of Thy-1N rats (in vivo), which might be some potential driving factors for SOX9 gene induction^61,62^. Although it is worth verifying whether these transcription factors are involved in SOX9 expression triggered by sublytic C5b-9, we think that it is more essential to disclose how ERK1/2 regulates SOX9 function based on the present data.

Posttranslational modifications (PTMs), e.g., phosphorylation, acetylation, and ubiquitination, play an important role in regulating protein function^63–65^. It has been pointed out that cAMP-dependent protein kinase A (PKA) phosphorylates SOX9 at the Ser64 and Ser181 residues, promoting its nuclear entry and transcriptional activation^47,66^, and cGMP-dependent protein kinase II (cGKII) or Rho-associated protein kinase 1 (ROCK1) phosphorylates SOX9 at Ser181^27^. In addition, our experiments found that sublytic C5b-9-activated ERK1/2 also phosphorylated SOX9 at the Ser181 site via a physical interaction in rat GMCs. Considering ERK1/2 nuclear entry after MEK1/2 activation^67^, we speculated that ERK1/2 might bind SOX9 in the GMC nucleus. As expected, the interaction between exogenous ERK1/2 and SOX9 was more evident in the cell nucleus, and ERK1/2 genetic knockdown or kinase inactivation reduced SOX9 expression and phosphorylation as well as its association with p-ERK1/2 in the GMC nucleus. However, at the initial stage, it is uncertain whether the SOX9/p-ERK1/2 complex is formed in the nucleus, leading to SOX9 retention, or is formed in the cytoplasm, promoting SOX9 nuclear translocation. Since ERK1/2-phosphorylated PKM2 at Ser37 enhances PKM2 interaction with cytoplasmic importin α5 and its translocation to the nucleus^68^, we hypothesized that SOX9 nuclear translocation might be regulated by ERK1/2 through a similar mechanism that needs to be demonstrated in the future.

In addition to phosphorylation, SOX9 acetylation can also alter its transcriptional activity^69^. Our previous data reflected that SOX9 could undergo acetylation by binding to KAT7, PCAF, or GCN5 in GMCs exposed to sublytic C5b-9 (data not shown). In this study, we proved that ERK1/2 also had an indirect effect on SOX9 acetylation induced by sublytic C5b-9. Our data showed that ERK1/2 activation facilitated SOX9 acetylation and its binding with KAT7, PCAF, and GCN5. Moreover, ERK1/2 determined cellular GCN5 protein levels. Recently, several reports have proven that SOX9 underwent SUMOylation or ubiquitination after phosphorylation by PKA at Ser64 and Ser181 or by glycogen synthase kinase 3β (GSK-3β) at Thr236^26,66^, indicating that SOX9 acetylation may be governed by ERK1/2 via either a phosphorylation-dependent or phosphorylation-independent mechanism. Thus, identification of SOX9 phosphorylation residues modified by ERK1/2 is necessary for further investigation.

Considering that the IB assay of SOX9 Ser181 alone cannot reflect the entire scope of SOX9 phosphorylation events driven by ERK1/2, we employed MS to detect SOX9 phosphorylation sites. Two phosphorylation sites, including the functional Ser64 residue and the novel Ser149 residue, were identified by MS. By a series of experiments in rat GMCs transfected with different phospho-mutant SOX9 plasmids, we demonstrated that ERK1/2 phosphorylation of SOX9 at the Ser64 and Ser181 residues was involved not only in SOX9 nuclear translocation but also in its acetylation, which enabled it to occupy the Cyclin D1 promoter, resulting in Cyclin D1 production and GMC proliferation. These data suggest that both SOX9 phosphorylation-independent and phosphorylation-dependent mechanisms are involved in SOX9 acetylation. From our perspective, there are at least four potential mechanisms: (a) ERK2 has been confirmed to phosphorylate p300, enhancing its autoacetylation and activity^70^ and this mechanism might also occur during ERK1/2 interaction with KAT7, PCAF and GCN5; (b) some HATs are mainly expressed in the nucleus^71,72^, which enables them to access and then acetylate SOX9; (c) our results showed that ERK1/2 can increase GCN5 protein; and (d) ERK1/2-phosphorylated SOX9 can recruit coactivators to chromatin and relax the compact chromatin structure, which is beneficial to gene transcription^73^. During this process, SOX9 may form a complex with some coactivators, which might trigger SOX9 acetylation afterwards.

Reportedly, acetylation of FOXO1 induced by CBP or that of FOXO3 induced by Sirt1/7 suppression is required for their further phosphorylation^74,75^, indicating that protein acetylation may also induce phosphorylation. We showed that although SOX9 acetylation had the same effect on Cyclin D1 expression and GMC proliferation, but this process did not affect its phosphorylation, suggesting that SOX9 phosphorylation by ERK1/2 occurs prior to but is necessary for its acetylation.

Our in vivo experiments showed that ERK1/2, SOX9, and cyclin D1 activation or expression exerted some influence on the Thy-1N rats, as knockdown of renal ERK1/2, SOX9, or Cyclin D1 gene expression reduced GMC proliferation and other lesions in Thy-1N rats. Consistent with our previous results, SOX9 phosphorylation, acetylation, and association with p-ERK1/2 were all decreased upon renal ERK1/2 gene silencing. In addition, we also verified the elevated expression of these proteins in the renal tissues of MsPGN patients and observed positive correlations between p-ERK1/2, p-SOX9, SOX9, and Cyclin D1 as well p-ERK1/2 and p-SOX9.

In summary, our study reveals that in the Thy-1N rats, sublytic C5b-9 stimulation of rat GMCs caused ERK1/2 activation via the calcium-PKC-α-c-Raf-MEK1/2 axis and upregulated the expression of SOX9 and Cyclin D1. The activated ERK1/2 then phosphorylated SOX9 at the Ser64 and Ser181 residues, enabling it to access the Cyclin D1 promoter region (−582 to −238 nt) and promoting Cyclin D1 gene transcription. Moreover, nuclear SOX9, which was elevated by phospho-ERK1/2, also undergoes acetylation via interaction with sublytic C5b-9-upregulated KAT7, PCAF, and GCN5, which might further enhance Cyclin D1 transcription and expression. This ERK1/2-SOX9-Cyclin D1 reaction cascade results in the GMC proliferative lesions of Thy-1N rats (Fig. 8f). Our findings on rat Thy-1N described above may provide a novel pathogenic mechanism of GMC proliferation and may indicate a series of therapeutic targets in human MsPGN.

## Supplementary information

Supplementary Figures
