# Supplementary material for: Sublytic C5b-9 induces glomerular mesangial cell proliferation via ERK1/2-dependent SOX9 phosphorylation and acetylation by enhancing Cyclin D1 in rat Thy-1 nephritis
Source: Exp Mol Med. 2021 Apr 2;53(4):572–90. doi: 10.1038/s12276-021-00589-9 (PMC8102557; doi:10.1038/s12276-021-00589-9)
Supplement: Supplementary file 1 — Supplementary Figures [file 12276_2021_589_MOESM1_ESM.docx]

**Supplementary Fig. S1**

**
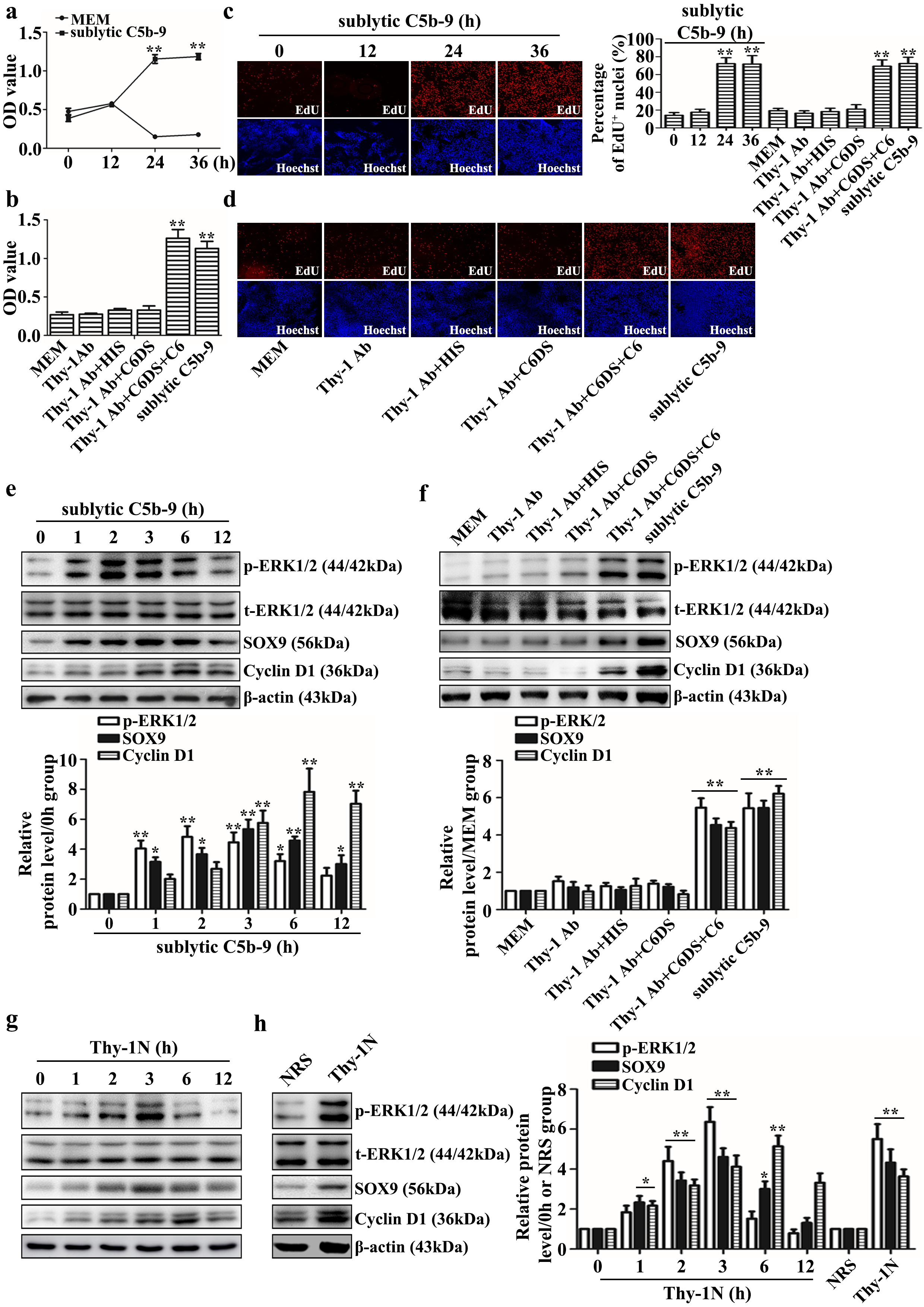
**

**Supplementary Fig. S1: GMC proliferation as well as the protein level of phosphorylated ERK1/2, SOX9 and Cyclin D1 in the GMCs treated with sublytic C5b-9 and renal tissues of Thy-1N rats. (a-d)** *In vitro* cultured rat GMCs were stimulated with sublytic C5b-9 for indicated time or with different treatments including MEM, Thy-1 Ab, Thy-1 Ab+HIS, Thy-1 Ab+C6DS, Thy-1 Ab+C6DS+C6, sublytic C5b-9 for 24h, GMC proliferation was determined by CCK-8 **(a, b)** and EdU incorporation assay **(c, d)**. ** p<0.01 versus MEM (24h/36h) or MEM, Thy-1 Ab, Thy-1 Ab+HIS, Thy-1 Ab+C6DS. **(e)** The protein level of t-ERK1/2, p-ERK1/2, SOX9 and Cyclin D1 in sublytic C5b-9-treated rat GMCs at different time points. **(f)** The protein level of p-ERK1/2, SOX9 and Cyclin D1 in GMCs at 3h in different groups. **(g)** The protein level of t- ERK1/2, p-ERK1/2, SOX9 and Cyclin D1 in the renal tissues of Thy-1N rats at different time points. **(h)** The protein level of t-ERK1/2, p-ERK1/2, SOX9 and Cyclin D1 at 3h in the renal tissues of Thy-1N and NRS control rats. * p<0.05, ** p<0.01 versus 0h, MEM, Thy-1 Ab, Thy-1 Ab+HIS, Thy-1 Ab+C6DS or NRS. Data are represented as means ± SD (n=5 in each group for CCK‐8 and EdU incorporation or *in vivo* or, n=3 *in vitro* in each time‐point or each group).

**Supplementary Fig. S2**

**
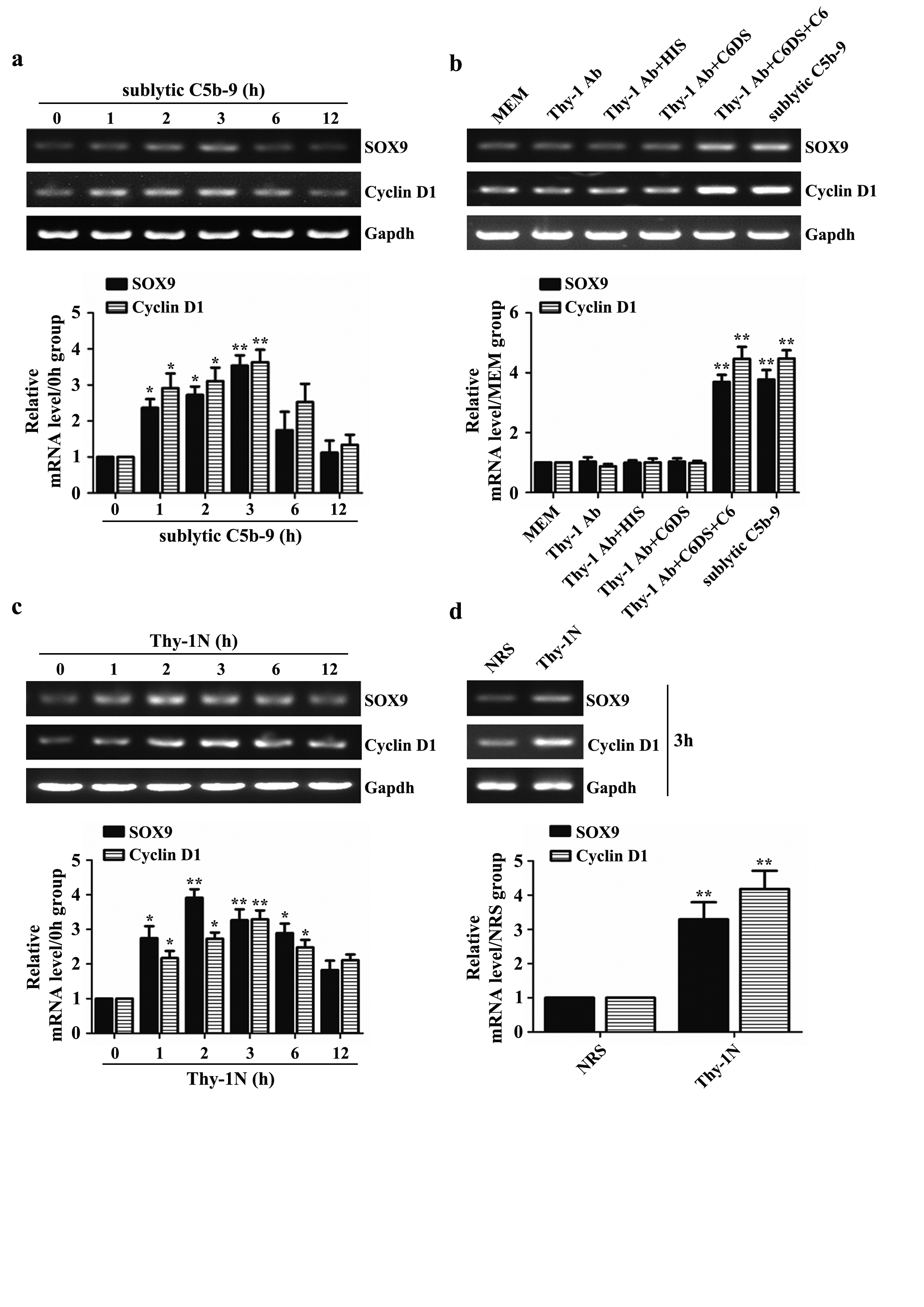
**

**Supplementary Fig. S2: The mRNA level of SOX9 and Cyclin D1 in the GMCs exposed to sublytic C5b-9 and the renal tissues of Thy-1N rats. (a)** The mRNA level of SOX9, and Cyclin D1 in sublytic C5b-9-treated GMCs at different time points. **(b)** The mRNA level of SOX9 (3h) and Cyclin D1 (3h) in different groups. **(c)** The mRNA level of SOX9 and Cyclin D1 in the renal tissues of Thy-1N rats at different time points. **(d)** The mRNA level of SOX9 (3h), and Cyclin D1 (3h) in the renal tissues of Thy-1N and NRS control rats. * p<0.05, ** p<0.01 versus 0h, MEM, Thy-1 Ab, Thy-1 Ab+HIS, Thy-1 Ab+C6DS or NRS group. Data are represented as means ± SD (n=3 *in vitro*, n=5 *in vivo* in each time‐point or each group).

**Supplementary Fig. S3
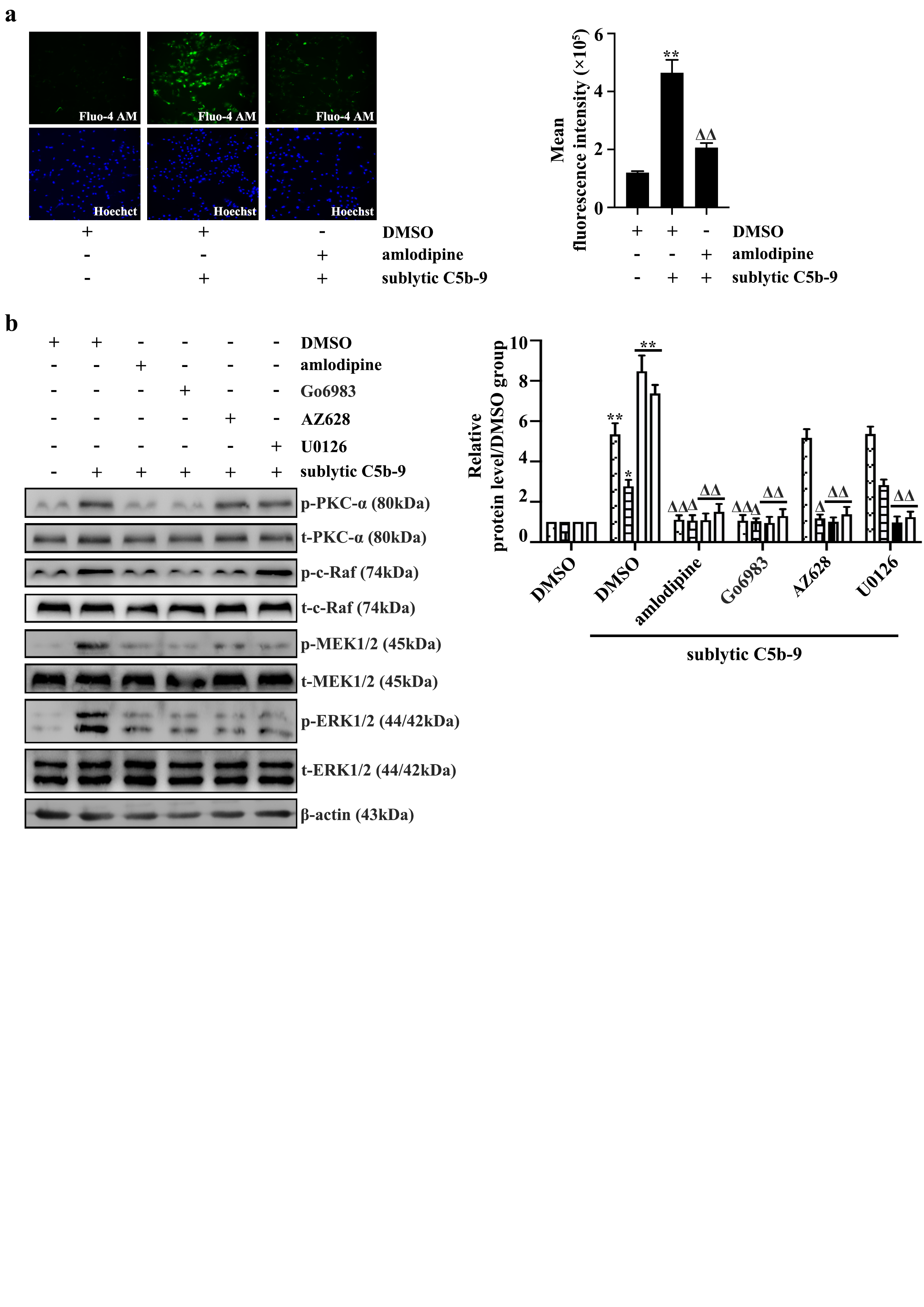
**

**Supplementary Fig. S3: Upstream pathway of ERK1/2 activation in the GMCs exposed to sublytic C5b-9.** Rat GMCs pretreated with the calcium channel blocker amlodipine (10μM), inhibitors of PKC (Go6983; 10μM), c-Raf (AZ628, 10μM) and MEK1/2 (U0126, 10μM) for 30min were stimulated with sublytic C5b-9 for 40min or 2h. The intracellular calcium was determined by Fluo-4 AM **(a)**. The protein phosphorylation and abundance of PKC-α, c-Raf, MEK1/2 and ERK1/2 were analyzed by IB **(b)**. * p<0.05, ** p<0.01 versus DMSO, ^Δ^ p<0.05, ^ΔΔ^ p<0.01 versus DMSO+sublytic C5b-9. Data are represented as means ± SD (n=3 in each group the other experiments).

**Supplementary Fig. S4**

**
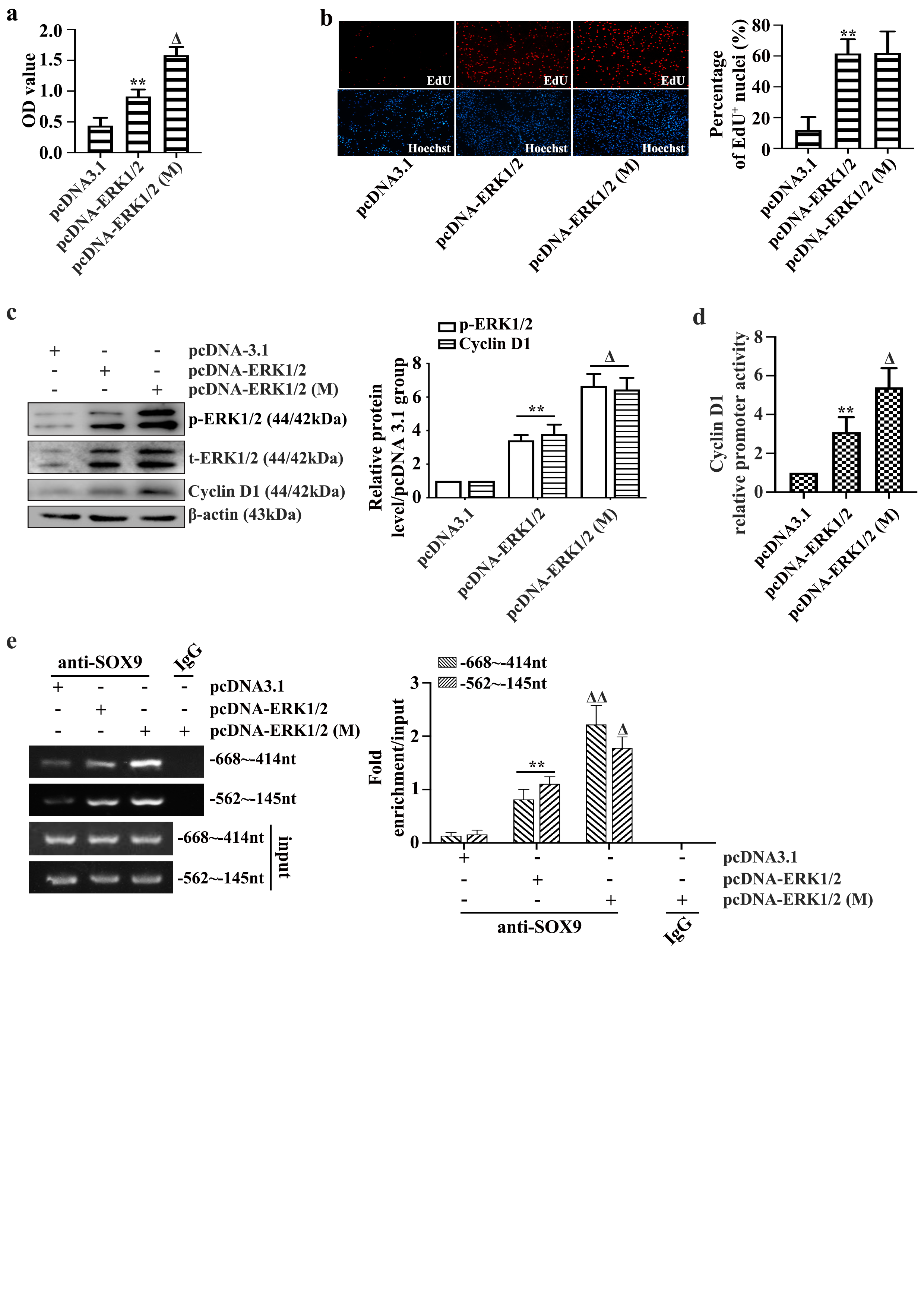
**

**Supplementary Fig. S4: The effect of ERK1/2 constitutively activation on GMC proliferation and Cyclin D1 gene induction. (a-e)** Rat GMCs were transfected with pcDNA-ERK1/2 or pcDNA-ERK1/2 (M) plasmids [pcDNA-ERK1 (R85S) + pcDNA-ERK2 (R65S)]. GMC proliferation was determined by CCK-8 and EdU incorporation assay **(a, b)**. Cyclin D1 protein abundance was analyzed by IB **(c)**. Cyclin D1 promoter activity was determined by luciferase reporter assay **(d)** and the recruitment of SOX9 to the Cyclin D1 promoter regions was tested by ChIP-PCR **(e)**. ** p<0.01 versus pcDNA3.1, ^Δ^ p<0.05, ^ΔΔ^ p<0.01 versus pcDNA-ERK1/2. Data are represented as means ± SD (n=5 in each group for CCK‐8 or EdU incorporation, n=3 in each group the other experiments).

**Supplementary Fig. S5**

**
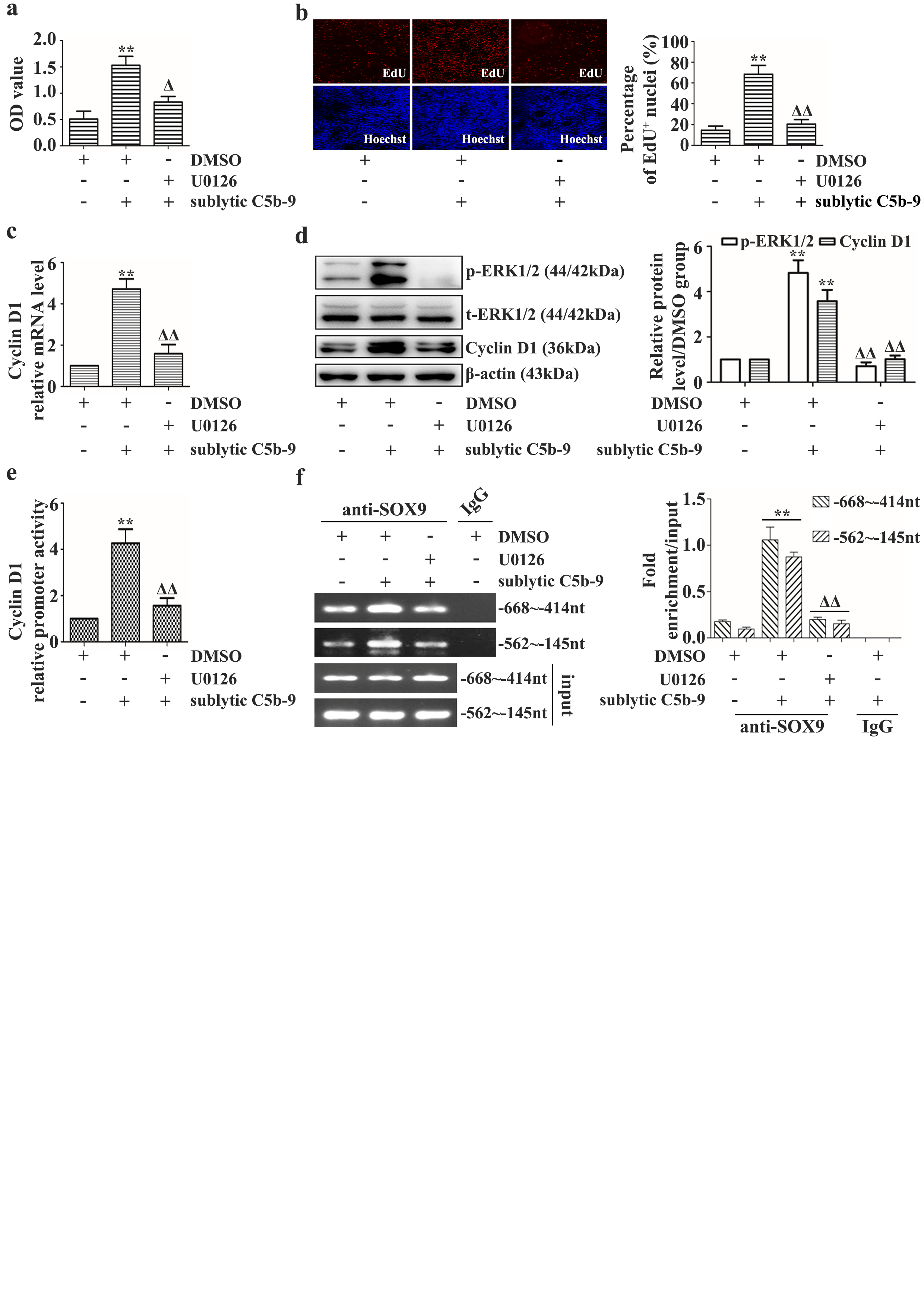
**

**Supplementary Fig. S5: The effect of ERK1/2 kinase inactivation on GMC proliferation and Cyclin D1 gene induction in response to sublytic C5b-9. (a-f)** Rat GMCs pretreated with the MEK inhibitor U0126 (10μM) for 30min were stimulated with sublytic C5b-9 for 24h or 3h. GMC proliferation was determined by CCK-8 and EdU incorporation assay **(a, b)**. The intracellular abundance of Cyclin D1 mRNA and protein was analyzed by qRT-PCR and IB **(c, d)**. Cyclin D1 promoter activity was determined by luciferase reporter assay **(e)** and the recruitment of SOX9 to the Cyclin D1 promoter regions was tested by ChIP-PCR **(f)**. ** p<0.01 versus DMSO, ^Δ^ p<0.05, ^ΔΔ^ p<0.01 versus DMSO+sublytic C5b-9. Data are represented as means ± SD (n=5 in each group for CCK‐8 or EdU incorporation, n=3 in each group the other experiments).

**Supplementary Fig. S6**

**
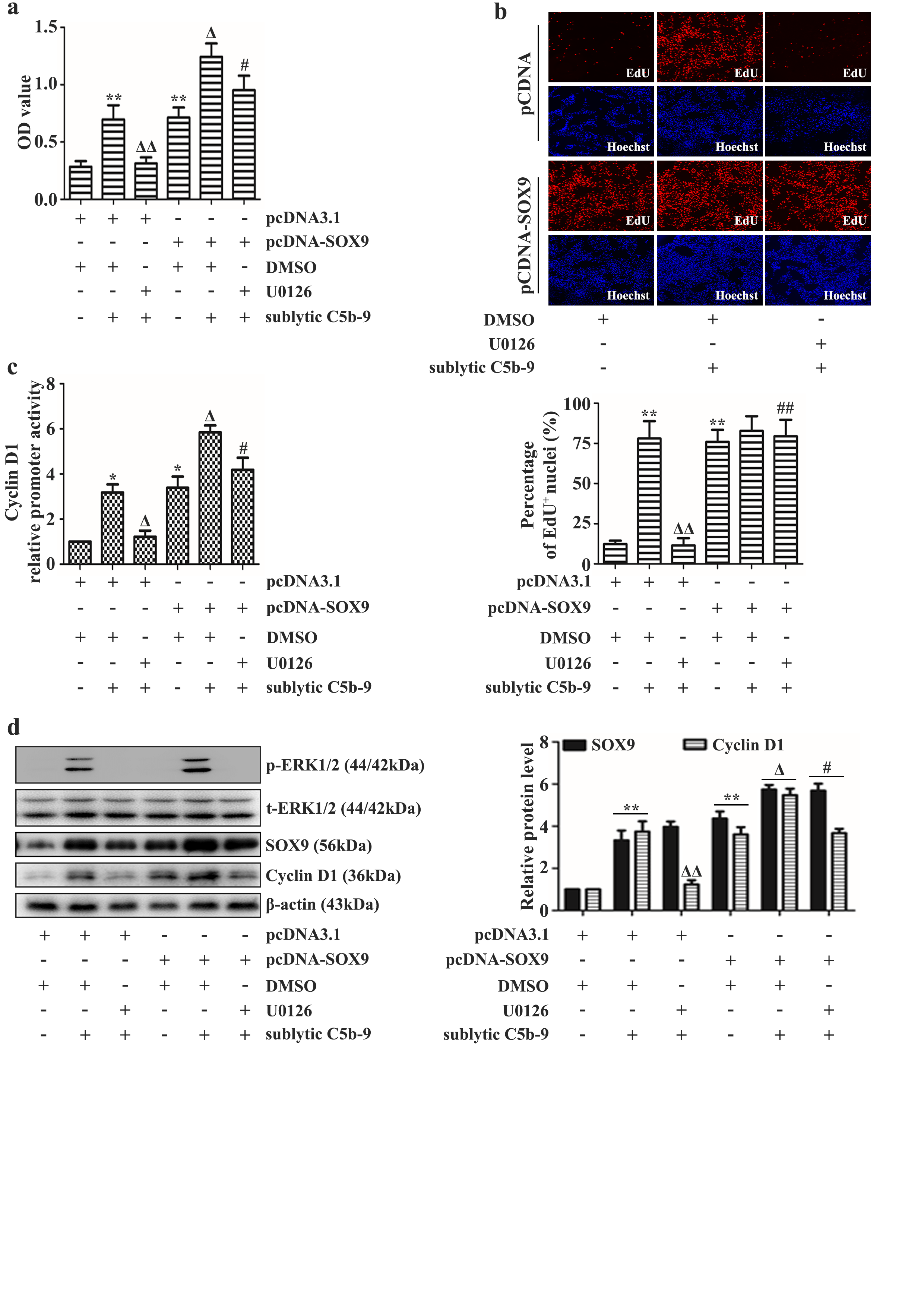
**

**Supplementary Fig. S6: Role of ERK1/2-SOX9 axis in sublytic C5b-9-exposed GMCs during cell proliferation and Cyclin D1 induction after U0126 treatment. (a-d)** Rat GMCs pre-transfected with pcDNA-SOX9 plasmid were then treated with U0126 (10μM) for 30min, followed by sublytic C5b-9 stimulation for 24h or 3h. GMC proliferation was determined by CCK-8 and EdU incorporation assay **(a, b)**. Cyclin D1 promoter activity and its protein level were analyzed by luciferase reporter assay **(c)** and IB assay **(d)**. * p<0.05 or ** p<0.01 versus pcDNA3.1+DMSO, ^Δ^ p<0.05 or ^ΔΔ^ p<0.01 versus pcDNA3.1+DMSO+sublytic C5b-9, ^#^ p<0.05 or ^##^ p<0.01 versus pcDNA3.1+U0126+sublytic C5b-9. Data are represented as means ± SD (n=5 in each group for CCK‐8 or EdU incorporation, n=3 in the other experiments).

**Supplementary Fig. S7**

**
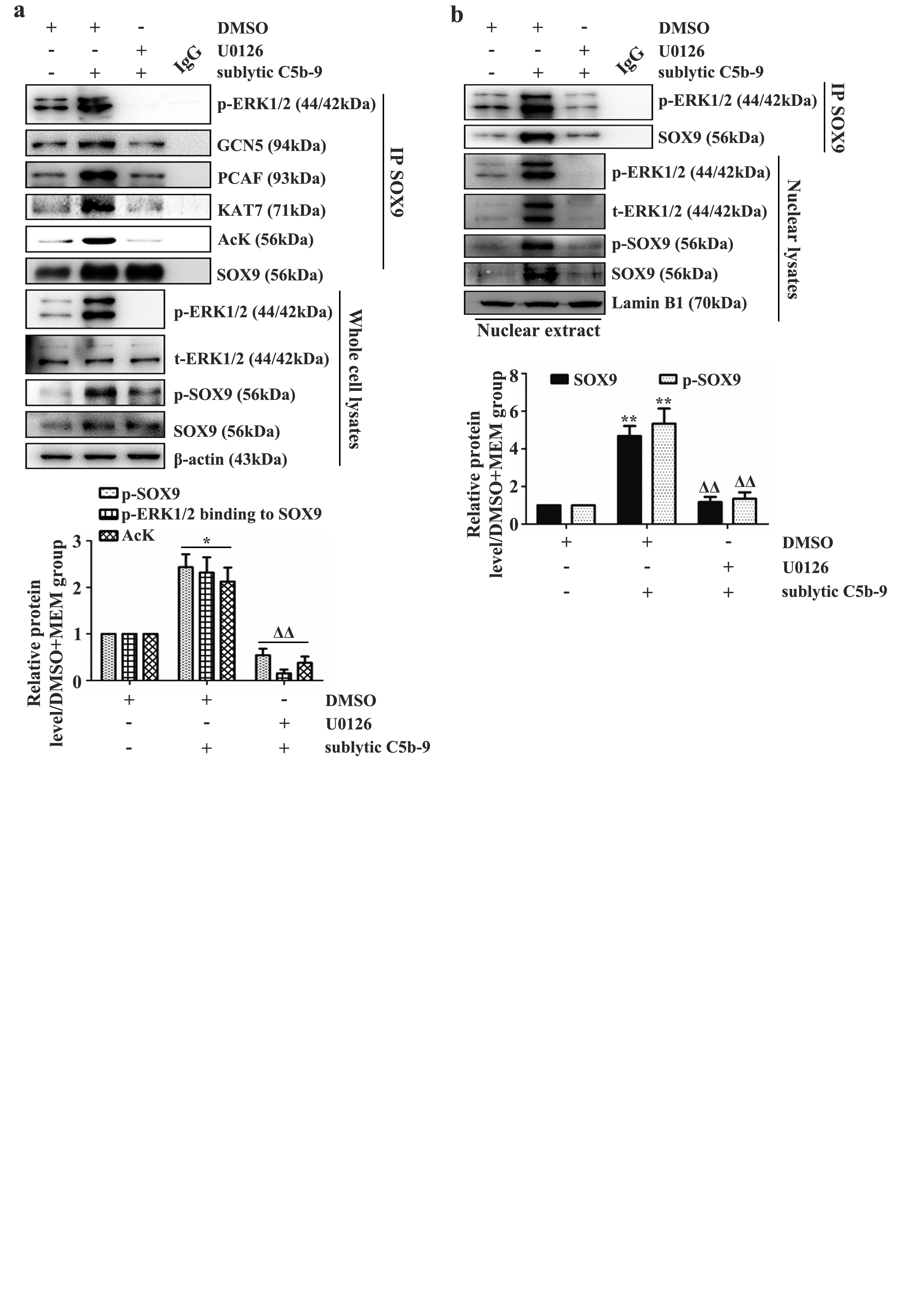
**

**Supplementary Fig. S7: The influence of** **ERK1/2 blockade on SOX9 phosphorylation, acetylation and nuclear expression in GMCs stimulated with sublytic C5b-9.** **(a)** Rat GMCs pretreated with U0126 (10μM) for 30min were stimulated with sublytic C5b-9 for 3h. SOX9 interaction with KAT7, PCAF, GCN5 and p-ERK1/2 and its acetylation level were measured by co-IP, and SOX9 phosphorylation level was detected by IB. **(b)** The nuclear component was isolated from the cells with the above-mentioned treatments, and SOX9 was immunoprecipitated followed by IB with SOX9 and p-ERK1/2 antibody. ** p<0.01 versus DMSO, ^ΔΔ^ p<0.01 versus DMSO+sublytic C5b-9 group. Data are represented as means ± SD (n=3 in each experiment).

**Supplementary Fig. S8**

**
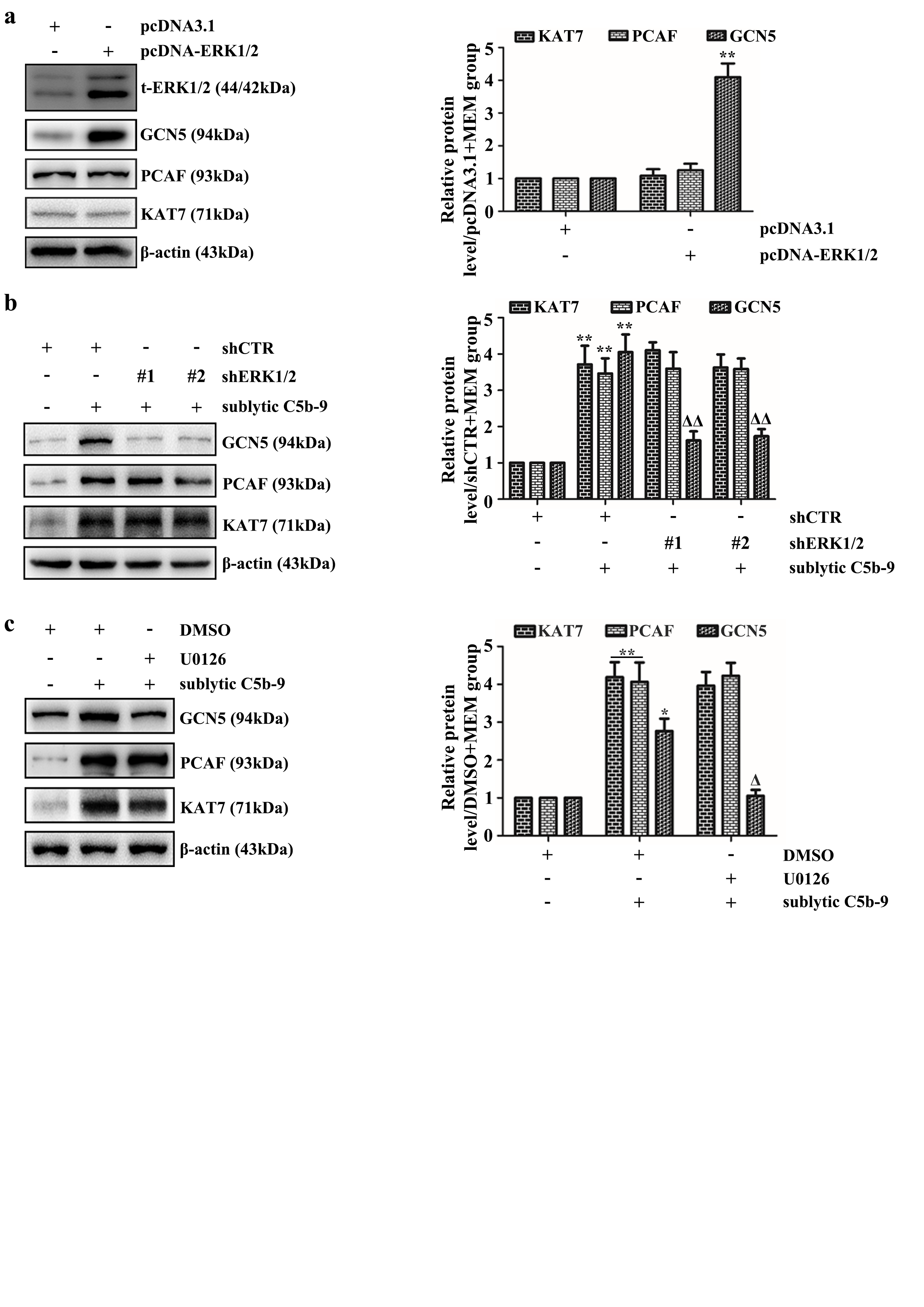
**

**Supplementary Fig. S8: The level of KAT7, PCAF and GCN5 in sublytic C5b-9-stimulated GMCs after ERK1/2 overexpression, knockdown or activity blockade. (a)** Rat GMCs transfected with pcDNA-ERK1/2 plasmid for 48h. **(b, c)** GMCs pre-transfected with shERK1/2 vector or pretreated with U0126 (10μM) were stimulated with sublytic C5b-9 for 3h. The protein level of t-ERK1/2, KAT7, PCAF and GCN5 was checked by IB. * p<0.05, ** p<0.01 versus pcDNA3.1, shCTR or DMSO, ^Δ^ p<0.05, ^ΔΔ^ p<0.01 versus shCTR+sublytic C5b-9 or DMSO+sublytic C5b-9. Data are represented as means ± SD (n=3 in each experiment).

**Supplementary Fig. S9**

**
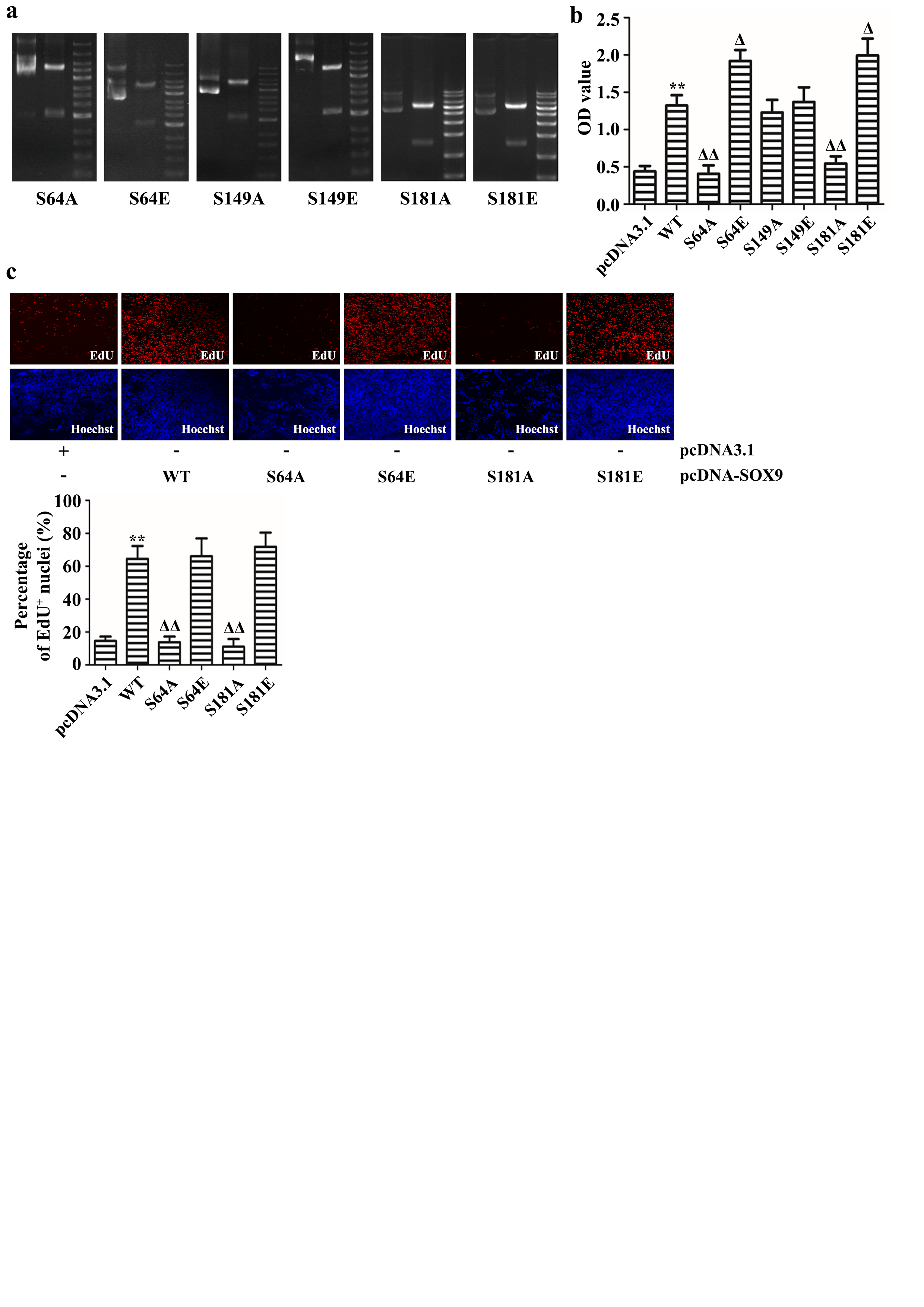
**

**Supplementary Fig. S9: GMC proliferative changes induced by SOX9 phosphorylation on Ser 64 and Ser 181. (a)** The phospho-mutant pcDNA-SOX9 plasmids (S64A/E, S149A/E and S181A/E) were generated. Left lane: naïve plasmid, middle: digested plasmid, right: DNA marker. **(b, c)** Rat GMCs were transfected with wild-type (WT) or different phospho-mutant pcDNA-SOX9 plasmids, cell proliferation was analyzed by CCK-8 **(b)** and EdU incorporation **(c)**, respectively. ** p<0.01 versus pcDNA3.1, ^Δ^ p<0.05, ^Δ^^Δ^ p<0.01 versus WT. Data are represented as means ± SD (n=5 in each group for CCK‐8 or EdU incorporation).

**Supplementary Fig. S10**


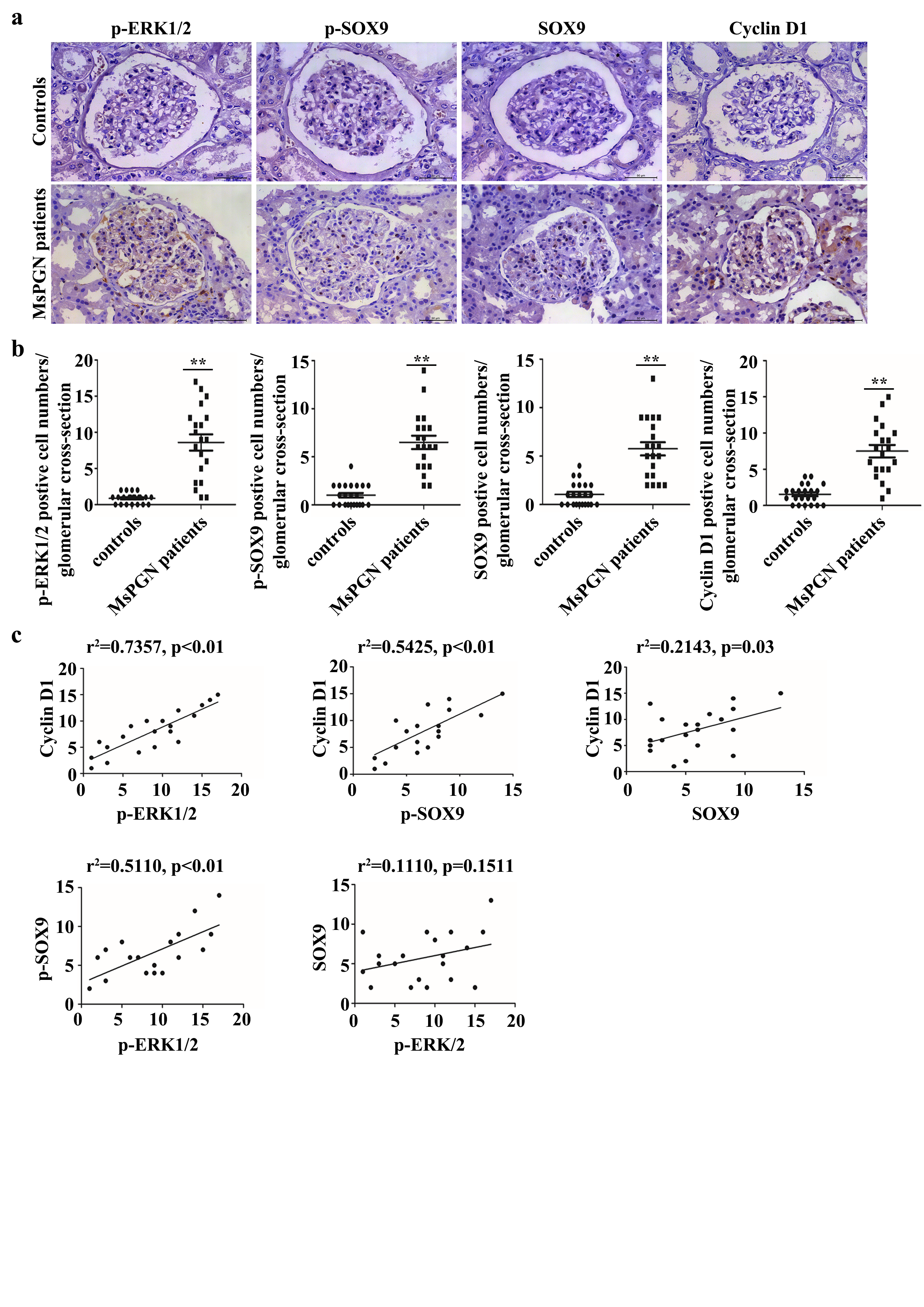


**Supplementary Fig. S10: Expression of p-ERK1/2, p-SOX9, SOX9 and Cyclin D1 in MsPGN patients. (a, b)** The protein expression of p-ERK1/2, p-SOX9, SOX9 and Cyclin D1 in the glomeruli of MsPGN patients (n=20) and controls (n=22) was examined using IHC staining (×400). **(c)** The correlation of these parameters was also analyzed. ** p<0.01 versus controls.

**Table S1. Specific primers used in PCR reaction (RT-PCR, qRT-PCR and ChIP-PCR), and plasmid construction.**

| **Plasmid/Gene** | **Primer** | **Sequence** |
| --- | --- | --- |
| SOX9 (RT-PCR) | Forward | GCCACCGAACAGACTCAC |
|  | Reverse | GTTGGGCGGCAGGTATTG |
| Cyclin D1 (RT-PCR) | Forward | CTGGAGCCCCTGAAGAAGA |
|  | Reverse | GGAGGGTGGGTTGGAAAT |
| Cyclin D1 (qRT-PCR) | Forward | AGAGGGAGATTGTGCCATCC |
|  | Reverse | ACAAGAACCGGTCCAGGTAG |
| Gapdh (RT-PCR) | Forward | CAAGGTCATCCATGACAACTTTG |
|  | Reverse | GTCCACCACCCTGTTGCTGTAG |
| β-actin (qRT-PCR) | Forward | TCACCCACACTGTGCCCATCTATGA |
|  | Reverse | CATCGGAACCGCTCATTGCCGATAG |
| -668 ~ -414nt (ChIP-PCR) | Forward | CGGTCACTGTAAGAAGGCAA |
|  | Reverse | ATGCCAGACGAGCCCTAA |
| -562 ~-145nt (ChIP-PCR) | Forward | TGAGATTTTCGGGGTTTT |
|  | Reverse | CCTGTGAAGGTGGGGTAG |
| -238 ~ -188nt (ChIP-PCR) | Forward | CCCCTTCCATACATTCTTTCTT |
|  | Reverse | CTCTGCTACTGCGCCAACA |
| pcDNA-Cyclin D1 | Forward | ctagcgtttaaacttaagcttCGCAGTAGCAGAGAGCTGCA |
|  | Reverse | aacgggccctctagactcgagATCCTTCTCAAGACTTCCTGTGTGT |
| pGL3-Cyclin D1 (full-length) | Forward | cgagctcttacgcgtgctagcGACTATAAATTTAAAATAGGCTTAGGGATC |
|  | Reverse | acttagatcgcagatctcgagACTCCCCTGTAGTCCGAGTGACG |
| pGL3-Cyclin D1 (-985~+130nt) | Forward | cgagctcttacgcgtgctagcAATGTCTTTAAATATCACCTTATCGGC |
|  | Reverse | acttagatcgcagatctcgagACTCCCCTGTAGTCCGAGTGACG |
| pGL3-Cyclin D1 (-582~+130nt) | Forward | cgagctcttacgcgtgctagcCCACCATCTTGAGCTGTTGCT |
|  | Reverse | acttagatcgcagatctcgagACTCCCCTGTAGTCCGAGTGACG |

The underlines represent restriction sites for digestion reactions.

**Table S2. Target sequences of indicated genes for shRNA construction.**

| **Gene** | **Sequence** |
| --- | --- |
| shCTR | TTCTCCGAACGTGTCACGT |
| shERK1-1  shERK1-2 | GGAAGCCATGAGAGATGTTTA  GCAATGACCACATCTGCTACT |
| shERK1-3  shERK1-4  shERK2-1  shERK2-2  shERK2-3  shERK2-4  shSOX9-1  shSOX9-2  shSOX9-3  shSOX9-4  shCyclin D1-1  shCyclin D1-2  shCyclin D1-3  shCyclin D1-4 | GGACCAGCTCAACCACATTCT  GGGCTACACCAAATCCATTGA  GGACCTCATGGAGACAGATCT  GCACCTCAGCAATGATCATAT  GCTGAACACCACTTGTGATCT  GCACCTCAGCAATGATCATAT  GGAGGAAGTCGGTGAAGAATG  GTGGATGTCAAAGCAACAGGC  GGAACAACCCGTCTACACACA  GAAGAAGGAGAGCGAGGAAGA  GCGAGCCATGCTTAAGACTGA  GCATGTTCGTGGCCTCTAAGA  GCAGATCATCCGCAAACATGC  GGAACAGATTGAAGCCCTTCT |
